# Split‐Standing Molecular Engineering for Textured Silicon/Perovskite Tandems

**DOI:** 10.1002/advs.202505288

**Published:** 2025-06-25

**Authors:** Xiaonan Wang, Yuan Tian, Libing Yao, Shaochen Zhang, Qingqing Liu, Ke Zhao, Jiazhe Xu, Jingjing Zhou, Caner Deger, Ilhan Yavuz, Jingjing Xue, Rui Wang

**Affiliations:** ^1^ State Key Laboratory of Silicon and Advanced Semiconductor Materials, School of Materials Science and Engineering Zhejiang University Hangzhou 310027 China; ^2^ Department of Materials Science and Engineering, School of Engineering Westlake University Hangzhou 310024 China; ^3^ Physics Department Marmara University Kadikoy Istanbul 34722 Turkiye

**Keywords:** anchoring stability, hole‐selective layers, indolocarbazole, inverted perovskite solar cells, textured silicon/perovskite tandem solar cells

## Abstract

To effectively minimize reflection losses and achieve compatibility with industrial‐scale silicon production lines, textured silicon/perovskite tandem solar cells have garnered significant attention in recent research. However, achieving uniform and stable coverage of the textured silicon substrate with hole‐selective layer (HSL) remains a significant challenge. Herein, a HSL material, DPAICz ((indolo[2,3‐a]carbazole‐11,12‐diylbis(ethane‐2,1‐diyl))bis(phosphonic acid)), is reported specifically designed for textured silicon substrate. Compared to the typical HSL material 2PACz, DPAICz features a π‐expanded conjugated core and multiple anchoring groups, forming a split‐standing configuration with anchoring groups positioned on opposite sides, resulting in superior anchoring stability on textured substrate under external stimuli. Moreover, DPAICz exhibited a larger molecular dipole moment and a more pronounced p‐type characteristic, enhancing the interfacial hole extraction efficiency. Consequently, wide‐bandgap (1.68 eV) perovskite solar cells employing DPAICz as the HSL achieved a champion power conversion efficiency (PCE) of 23.42%. Introducing the DPAICz into monolithic silicon/perovskite tandem solar cells greatly improved their performance, achieving a remarkable PCE of 32.55% in 1 cm^2^ area. Importantly, the unencapsulated tandems based on DPAICz exhibited significantly enhanced long‐term operational stability, retaining 96% of its initial PCE after 880 h of continuous 1‐sun light soaking at 45 °C under open‐circuit condition.

## Graphical abstract

An indolocarbazole‐based HSL material (DPAICz) with a split‐standing molecular configuration is reported specifically designed for textured silicon substrates. Due to its strong anchoring stability and excellent hole extraction efficiency, monolithic textured silicon/perovskite tandem solar cells employing DPAICz as the HSL achieved a remarkable power conversion efficiency of 32.55% in 1 cm^2^ area, along with significantly enhanced long‐term operational stability.

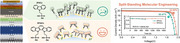

## 1 Introduction

Tandem perovskite solar cells, combining perovskite materials with other solar cell technologies, have emerged as a promising strategy to break through the theoretical efficiency limits of single‐junction perovskite solar cells.^[^ ^1^, ^2^, ^3^ ^]^ In tandem solar cells, the hole selective layer (HSL) in the perovskite top cells, which can facilitate efficient charge transport, reduce recombination losses, and provide a protective barrier for the perovskite active layer, plays a crucial role in optimizing device performance and stability.^[^ ^4^, ^5^, ^6^, ^7^, ^8^, ^9^ ^]^ For achieving higher power conversion efficiency (PCE), monolithic silicon/perovskite tandem solar cells are typically constructed on double‐textured silicon heterojunction cells.^[^ ^2^, ^10^, ^11^, ^12^ ^]^ However, the small molecule‐based HSL materials commonly used in textured silicon tandems present certain limitations. Specifically, the textured surface requires HSL materials with stronger anchoring groups to ensure uniform and stable coverage of the pyramids.^[^ ^13^, ^14^, ^15^ ^]^ Commonly used HSL material, such as 2PACz, typically possesses only one anchoring group, which leads to poor stability and increased susceptibility to degradation under operating conditions, ultimately compromising the long‐term reliability of the whole device.^[^ ^7^, ^16^, ^17^ ^]^ Furthermore, the dipole moment orientation of 2PACz is suboptimal, negatively affecting its efficiency in hole extraction.^[^ ^18^, ^19^, ^20^ ^]^ Therefore, the development of HSL materials with enhanced anchoring strength, improved stability, and superior hole extraction efficiency is essential for optimizing both the performance and long‐term durability of tandem solar cells.

Here, we report the design and synthesis of a bipedal indolocarbazole‐based HSL material for textured silicon, DPAICz ((indolo[2,3‐a]carbazole‐11,12‐diylbis(ethane‐2,1‐diyl))bis(phosphonic acid)), which exhibits enhanced anchoring strength and superior hole extraction efficiency. Compared to 2PACz, DPAICz features a π‐expanded conjugated core and multiple anchoring groups. These structural modifications facilitate a split‐standing configuration, with two anchoring groups positioned on opposite sides of the π‐conjugated core, significantly enhancing the binding strength between the HSL and ITO. This improvement increases the material's resistance to external stimuli, thereby extending the service life of the whole device. Experimental results demonstrated that the DPAICz‐based HSL films exhibited superior stability after solvent rinsing and heat treatment, both on flat glass substrate and textured silicon substrate. Furthermore, the split‐standing molecular design leads to a larger dipole moment of 2.5 D in DPAICz, with the dipole orientation aligned perpendicular to the substrate, which significantly improving the hole extraction efficiency of the HSL material. By utilizing DPAICz as the HSL, the single junction wide‐bandgap perovskite solar cells (PSCs) of 1.68 eV obtained a champion PCE of 23.42%, an open‐circuit voltage (*V*_OC_) of 1.25 V, and a fill factor (FF) of 86.67%. Transferring the single‐junction improvements into tandem devices allowed us to fabricate monolithic silicon/perovskite tandem solar cells with a champion PCE of 32.55%.

## 2 Results and Discussion

### 2.1 Poor Stability of 2PACz on ITO and Molecule Design for Textured Silicon

**Figure 1:**
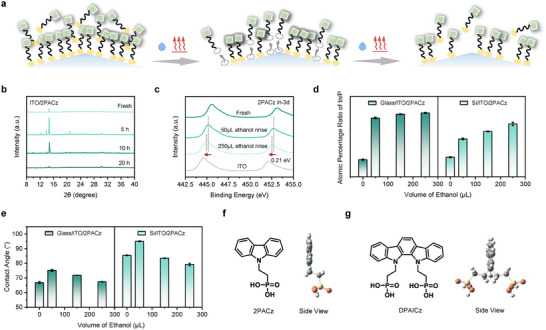
Degradation mechanism of 2PACz and molecular design. a) Schematic illustration of degradation of 2PACz on textured silicon/ITO surface under external stimuli (solvent rinse/heat). b) XRD patterns of 2PACz on ITO during 20 h at 150 °C heating conditions. c) XPS spectra of In 3d core level for 2PACz deposited onto Glass/ITO substrate rinsed by increasing volume of ethanol. d) Atomic percentage ratio of In/P on different substrates deposited with 2PACz after rinsing with different volume of ethanol. e) Contact angle of water for 2PACz deposited on different substrates after rinsing with different volume of ethanol. Molecular structure and side view of f) 2PACz and g) DPAICz.

Organic small molecules used as hole‐selective layers typically consist of three parts: an anchoring group, a spacer, and a conjugated terminal group.^[^ ^21^, ^22^, ^23^, ^24^ ^]^ Under the synergistic effect of the anchoring group and the spacer, the π–π interactions between the conjugated terminal groups induce stacking of molecules, enabling them to self‐assemble into periodic structures on the substrate. The typical HSL material, 2PACz, possesses only a single anchoring group, rendering its periodic structure vulnerable to disruption when exposed to external stimuli such as solvent rinse or heat, which will lead to film degradation.^[^ ^7^, ^17^ ^]^ The degradation mechanism of 2PACz under external stimuli was illustrated in **Figure** **1**a. The unstable stacking modes of 2PACz under external thermal stress were revealed by X‐ray diffraction (XRD) measurements (Figure 1b, the specifics are presented in Figure S1, Supporting Information). The distinct diffraction patterns of the 2PACz film confirmed the formation of long‐range ordered π‐π stacking. The fresh 2PACz film exhibits a main diffraction peak at 14.7°, accompanied by weak diffraction patterns centered at 9.8° and 21.3°, suggesting multiple molecular stacking modes. After heating at 150 °C for 5 h, several new diffraction peaks appeared at 12.3°, 13.9°, and 20.8°. With continued heating, the main diffraction peak at 14.7° weakened, broadened, and became asymmetric. After 20 h, the characteristic diffraction signals of 2PACz disappeared, indicating the severe disruption of the ordered stacking configuration of the 2PACz molecules.

The poor anchoring stability of 2PACz under solvent rinsing conditions was also investigated by high resolution X‐ray photoelectron spectroscopy (HR‐XPS) and contact angle technologies. Through the dynamic spin‐coating process, we used ethanol for rinsing different substrates deposited with 2PACz. As the volume of solvent increased, the In‐3d and Sn‐3d core level peaks for the 2PACz‐modified Glass/ITO substrate experienced a downward energy shift of 0.21 eV (Figure 1c and Figure S2, Supporting Information), suggesting the desorption of 2PACz from the flat glass substrate under solvent rinsing. In tandem devices, double‐textured silicon with a mildly textured front surface was used to achieve higher PCE. On such a rough substrate, the anchoring stability of 2PACz is deteriorated further. As observed, the In‐3d peak of the textured silicon/ITO substrate shifted 0.26 eV (Figure S3, Supporting Information) toward lower binding energy with increasing volume of solvent, indicating the more severe desorption of 2PACz on the textured substrate compared to the flat one.

We also performed a semi‐quantitative analysis of the elemental contents by HR‐XPS to examine changes in the content of anchored 2PACz on different substrates under solvent rinsing conditions. The coverage of the ITO surface can be reflected by the ratio of the characteristic element P in the HSL material to the characteristic element In in ITO.^[^ ^19^ ^]^ As shown in Figure 1d, the atomic percentage ratio of In/P on both substrates modified with 2PACz exhibited a sharp increase in the initial stage, which is attributed to the detachment of the unanchored multiple‐layer structures from the ITO. This is consistent with the results of the contact angle test (Figure 1e). Compared to the fresh film, the contact angle of the 2PACz‐modified substrate increased after rinsing with a small amount of solvent. This indicates that the disordered, unanchored molecules at the top of the multiple layers were washed away, leaving a more compact HSL with an orderly arrangement, where the hydrophobic conjugated terminal groups are oriented upward. Subsequently, the continuing rise of the In/P ratio with increasing solvent volume originated in the desorption of anchored 2PACz from the ITO surface. During this stage, the contact angle of the 2PACz‐modified substrate also decreased continuously (Figure 1e), indicating increased exposure of the ITO surface. This further confirmed that 2PACz molecules anchored to the ITO were easily desorbed under solvent stimuli. Additionally, compared to the 7° reduction in the contact angle on the Glass/ITO substrate (from 74.6° to 67.6°), a more significant decrease of 15.8° in the contact angle was observed on the textured silicon/ITO substrate (from 95.0° to 79.2°), demonstrating the weaker anchoring stability of 2PACz on the textured silicon surface once again (Figures S4, S5, Supporting Information).

In light of this, we proposed a new HSL material for textured silicon/perovskite tandem solar cells, DPAICz ((indolo[2,3‐a]carbazole‐11,12‐diylbis(ethane‐2,1‐diyl))bis(phosphonic acid)), featuring a π‐expanded conjugated core and multiple anchoring groups (Figure 1g). We addressed the issue of poor anchoring stability by increasing the number of anchoring groups. Simultaneously, by introducing a non‐coplanar screw‐shaped indolo[2,3‐a]carbazole unit as the core and a molecular structure with two anchoring groups positioned on opposite sides of the π‐conjugated core in a split‐standing configuration, we successfully optimized the dipole moment and enhanced the hole extraction efficiency of HSL, ultimately improved the efficiency and stability of the whole devices.

### 2.2 Split‐standing Molecular Engineering: Enhanced Resistance to External Stimuli

**Figure 2:**
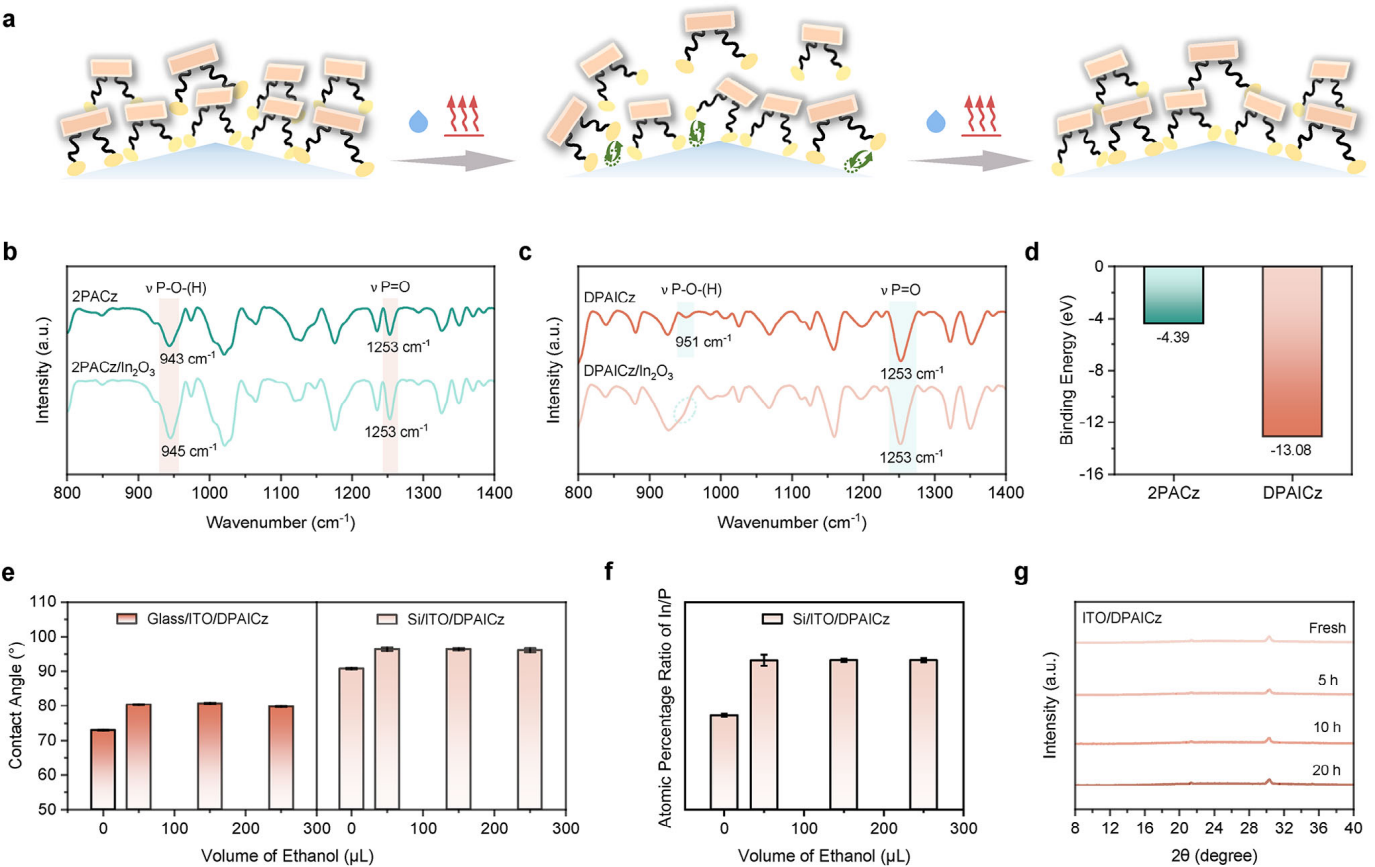
Anchoring mechanism and stability studies of DPAICz. a) Schematic illustration of adsorption of DPAICz on textured silicon/ITO surface under external stimuli (solvent rinse/heat). FTIR spectrum of b) 2PACz powder and mixed powder of In_2_O_3_/2PACz and c) DPAICz powder and mixed powder of In_2_O_3_/DPAICz. d) Calculated binding energy of 2PACz and DPAICz to the ITO surface. e) Contact angle of water for DPAICz deposited on different substrates after rinsing with different volume of ethanol. f) Atomic percentage ratio of In/P on the surface of textured silicon/ITO substrate deposited with DPAICz after rinsing with different volume of ethanol. g) XRD patterns of DPAICz on ITO during 20 h at 150 °C heating conditions.

The deposition and desorption mechanism of DPAICz on the ITO surface under external stimuli was illustrated in **Figure** **2**a. With two anchoring groups enabling robust adsorption on the ITO surface, DPAICz, even under external stimuli causing the dissociation of one phosphate group, can rely on the remaining phosphate group to maintain its binding to the ITO surface, allowing the bipedal‐anchoring configuration to be restored during subsequent processes, thereby leading to a stable HSL. The stronger combination between DPAICz and ITO was identified with the Fourier‐transform infrared (FTIR) spectroscopy. Since the poor signal‐to‐noise ratio and resolution in critical spectral regions of infrared reflection‐absorption spectroscopy (IRRAS) spectra obtained using FTIR system on the modified ITO/silicon surface, we collected the attenuated total reflection (ATR) spectra for 2PACz and DPAICz powders, as well as for powders prepared by mixing each compound with indium oxide in stoichiometric ratios (Figure 2b,c). The peaks for the P = O vibrations in the 1253 cm^−1^ region were observed in both spectra and no changes in the relative intensity or placement of the large *ν* (P = O) peaks were detected after 2PACz and DPAICz molecules interacted with indium oxide powder, suggesting the classic bidentate binding mode of the phosphonic acid of these two HSL materials, in which free P = O moieties are left unattached.^[^ ^25^ ^]^ The characteristic peaks between ca. 940 and 960 cm^−1^ are associated with P‐O‐(H) stretching vibrations, and their intensities are closely associated with the deprotonation of the phosphonic acid groups.^[^ ^26^ ^]^ After interacting with indium oxide, the P‐O‐(H) stretching vibration signals of 2PACz remained visible and exhibited a blue shift of 2 wavenumbers. The intensity of the P‐O‐(H) stretching vibration peak of DPAICz significantly decreased following the interaction of DPAICz and indium oxide, suggesting that the majority of DPAICz molecules had bound to indium oxide. This demonstrated that the deprotonation capability of the phosphonic acid groups in DPAICz is enhanced, leading to a stronger combination between DPAICz and ITO. This can be further confirmed by the larger changes in the binding energies of the In‐3d core level and Sn‐3d core level of DPAICz film deposited on the ITO substrate than the 2PACz film, as obtained by HR‐XPS spectra (Figure S6, Supporting Information). The stronger combination between DPAICz and ITO was also supported by the first principles calculations. The results showed that, two anchoring groups in DPAICz positioned on opposite side of the π‐conjugated core while anchoring to the ITO surface, whereas 2PACz anchored only with a single group (Figure S7, Supporting Information). The calculated binding energies of 2PACz and DPAICz on ITO surface are −4.39 eV and −13.08 eV (Figure 2d), respectively, indicating a more stable configuration for DPAICz, with two phosphonic acid groups anchored to the ITO surface.

Next, we investigated the anchoring stability of DPAICz under solvent rinsing conditions by atomic force microscope (AFM) technologies, HR‐XPS, and contact angle. We applied scanning Kelvin probe force microscopy (KPFM) to record the surface potential evolution of flat Glass/ITO substrate modified with 2PACz and DPAICz after solvent rinsing. As shown in Figures S8–S10 (Supporting Information), the contact potential distribution (CPD) of both 2PACz‐coated‐ITO and DPAICz‐coated‐ITO changed after the first solvent rinsing process, which can be attributed to the detachment of unanchored molecules from the upper layers of the HSL. With increasing volume of rinsing solvent, the CPD of 2PACz‐coated‐ITO decreased from −446.8 mV to −375.0 mV (Table S1, Supporting Information), providing evidence for further exposure of the ITO surface as a portion of anchored 2PACz molecules were washed away. In contrast, the CPD of DPAICz‐coated‐ITO remained nearly unchanged under further rinsing process, indicating that DPAICz molecules adsorbed on the Glass/ITO surface are highly resistant to desorption, demonstrating the superior anchoring stability of DPAICz. This was also supported by HR‐XPS results, which showed that the amount of rinsing solvent caused no shifts in the In‐3d core level peaks and Sn‐3d core level peaks of the flat Glass/ITO substrate deposited with DPAICz (Figure S11, Supporting Information). This behavior contrasts with the previously discussed 2PACz‐modified substrate, in which the signals of In‐3d and Sn‐3d core level peaks shifted from higher binding energies toward those observed in bare ITO as solvent rinsing progressed. Moreover, as the amount of rinsing solvent increases, the unchanged binding energy of In element in the DPAICz‐modified textured silicon/ITO substrate validated the excellent anchoring stability of DPAICz on textured silicon surface (Figure S12, Supporting Information). This was consistent with the results of the contact angle test (Figure 2e). Apart from the initial increase in the contact angle caused by the removal of unanchored molecules from the ITO surface via rinsing with a small amount of solvent, the contact angles of DPAICz‐modified substrate remained stable with subsequent rinsing both on flat ITO and textured silicon (Table S2 and Figure S13, S14, Supporting Information).

The superior anchoring stability of DPAICz compared to 2PACz on the textured silicon surface is further supported by the results of a semi‐quantitative elemental analysis via HR‐XPS. As shown in Figure 2f, the In/P ratio on the DPAICz‐modified textured silicon/ITO substrate remained constant throughout the rinsing. In contrast, according to the previous experimental results, the In/P ratio of the 2PACz‐coated textured silicon/ITO substrate increased continuously during the rinsing process, indicating persistent desorption of 2PACz from the surface of textured silicon/ITO. The variation in contact angles of 2PACz‐ and DPAICz‐modified textured silicon/ITO substrates during the solvent rinsing process further supports this observation (Figure S15, Supporting Information). Therefore, it can be concluded that, compared to 2PACz, DPAICz can maintain stable anchoring on both flat Glass/ITO and textured silicon/ITO surfaces under solvent rinsing stimuli.

We also conducted XRD measurements to examine the stacking stability of DPAICz under external thermal stress. It is observed that the fresh DPAICz film does not exhibit any diffraction signals (Figure 2g), indicating that DPAICz forms an amorphous structure on the ITO surface rather than adopting a long‐range ordered π–π stacking, as seen in the case of 2PACz. Polarized Raman spectroscopy results showed that 2PACz exhibited polarization‐dependent variations in Raman peak intensity, whereas the peak intensity corresponding to the C ═ C stretching in the π‐conjugated core of the DPAICz film was almost independent of the polarization angle (Figure S16, Supporting Information), which is a typical feature of amorphous structures with random molecular packing. This can be easily explained by the positioning of the two anchoring groups of DPAICz on opposite sides of the conjugated core, which increases the distance between π‐π interactions. Furthermore, the density functional theory (DFT) optimized structure reveals that the conjugated core of DPAICz adopts a slightly twisted structure, with a dihedral angle of 4.59° between the central benzene ring and the lateral indole nucleus on either side (Figure S17, Supporting Information), resulting in a helical structure that further hinders the capability of DPAICz to form ordered assemblies on the substrate. Notably, after subsequent heat treatment, the XRD pattern of DPAICz still shows no diffraction signals, suggesting that the DPAICz molecules on the ITO surface maintain this amorphous stacking structure under external thermal stress^[^ ^27^ ^]^.

**Figure 3:**
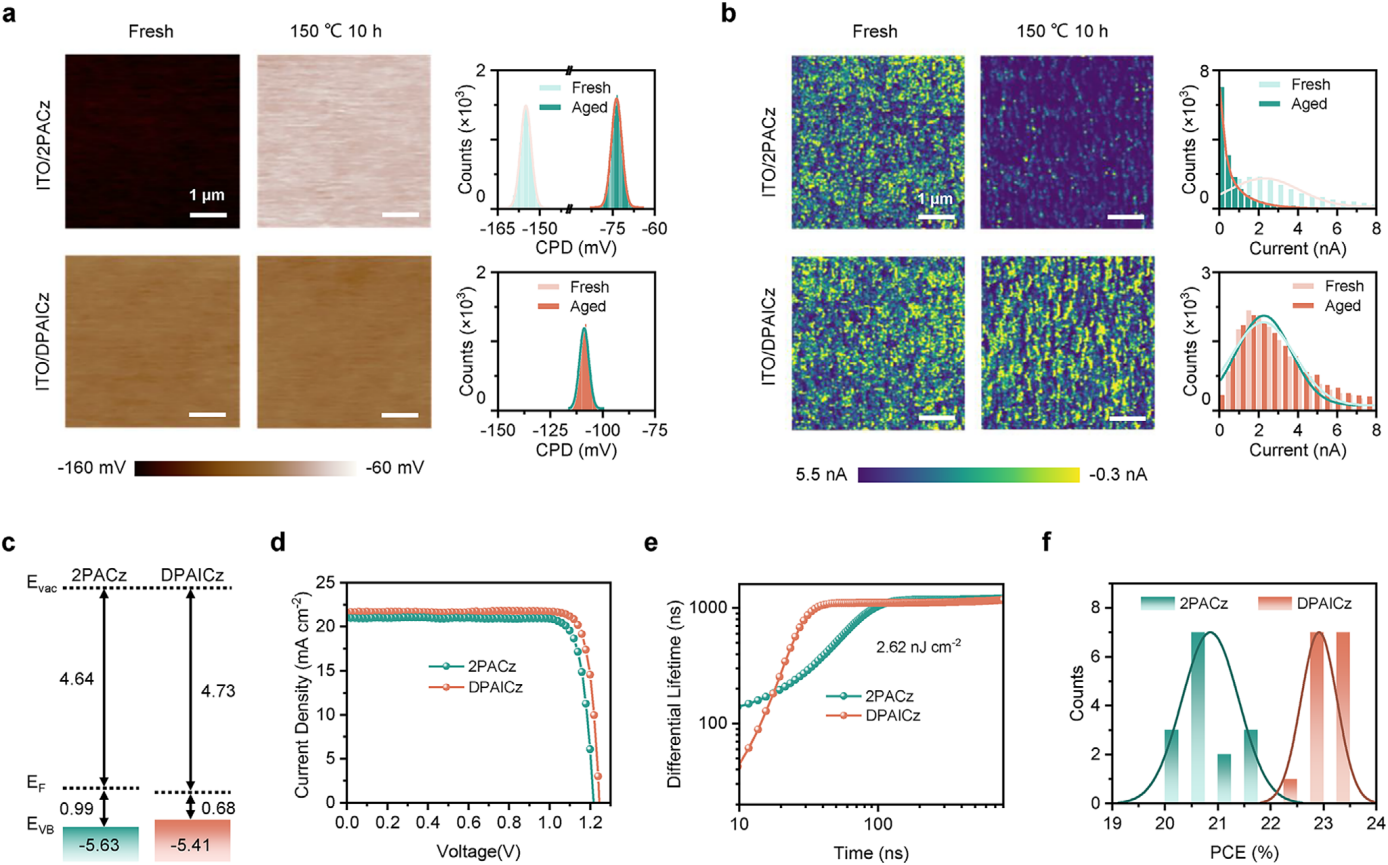
Electronic properties of the molecular films and single‐junction PSCs performance. a) Surface potential images obtained from scanning Kelvin probe force microscopy (KPFM) of the HSL before and after aging at 150 °C for 10 h. At the right of the figures are the statistical potential distributions of the film surfaces. b) Conductive atomic force microscopic (c‐AFM) images of the HSL before and after aging at 150 °C for 10 h. At the right of the figures are the statistical surface current signals of the film surfaces. c) Schematic representation of the band edge positions of the studied HSL based on values from UPS measurements, referenced to the vacuum level. d) *J*–*V* curves of wide band‐gap perovskite solar cells based on 2PACz and DPAICz. e) Differential lifetime derived from TRPL measurements at a low laser fluence of 2.6 nJ cm^−2^ for perovskite films in contact with 2PACz and DPAICz. f) Statistics of PCE values of wide band‐gap perovskite solar cells based on 2PACz and DPAICz.

To estimate the thermal stability of the ITO/DPAICz substrate, we applied KPFM and conductive atomic force microscopic (c‐AFM) to record the surface potential and surface current evolution of the HSL under heat treatment by taking ITO/2PACz as a reference. Based on the KPFM results (**Figure** **3**a), before thermal aging, both ITO/2PACz and ITO/DPAICz substrates exhibited relatively uniform surface potential with a narrow contact potential distribution of ≈15 mV, suggesting dense packing of the HSL materials on the ITO surface. After aging at 150 °C for 10 h, the CPD of the ITO/DPAICz substrate remained virtually unchanged. In contrast, the CPD of the ITO/2PACz substrate increased by 70 mV, consistent with previous reports, we attribute to the desorption, crystallization morphology changes under thermal stress, or a combination of both. According to c‐AFM results (Figure 3b), the averaged surface current signals of fresh ITO/2PACz and ITO/DPAICz substrates were 2.08 nA and 2.35 nA, respectively. The expanded π conjugated core design of DPAICz increases the degree of conjugation, resulting in higher conductivity. After aging at 150 °C for 10 h, the surface current signals of the ITO/2PACz substrate decreased significantly, while the surface current signals of the ITO/DPAICz substrate showed negligible change. Considering the preceding characterization results, we can conclude that the ITO/DPAICz substrate has superior resistance to external stimuli, including solvent rinsing and thermal treatment, while the ITO/2PACz film undergoes significant degradation under the same conditions.

### 2.3 Superior Hole Extraction Efficiency of DPAICz and Photovoltaic Performances of Single‐junction PSCs

Next, we performed ultra‐violet photoelectron spectroscopy (UPS) to assess the band characteristics of the HSL (Figure S18, Supporting Information), and the results were summarized in Figure 3c. The DPAICz‐modified substrate exhibited a down shift of the work function compared to the 2PACz‐modified substrate, which can be attributed to the increased dipole moment of DPAICz. According to the DFT calculations (Figure S19, Supporting Information), the DPAICz has a larger dipole moment than 2PACz (2.5 D vs 1.9 D), and its dipole moment is oriented perpendicular to the ITO substrate due to the two anchoring groups positioned on opposite sides of the conjugated core. In contrast, the dipole moment of the 2PACz, with a single anchoring group, is tilted toward the ITO substrate, resulting in a smaller component of the dipole moment pointing perpendicular to the ITO surface. Although some studies have reported the occurrence of depolarization when polar molecules are used to modify substrates, ^[^ ^28^, ^29^ ^]^ in the present work, the HSL materials chemically anchored to the ITO via phosphate groups, exhibiting a uniform orientation with conjugated groups facing upward and a stable anchoring mode. This configuration eliminates the occurrence of depolarization caused by antiparallel orientation of polar groups and conformational changes. Moreover, for the UPS onset, the DPAICz film showed a value of 0.68 eV, while the 2PACz exhibited a higher value of 0.99 eV, indicating the HSL derived from DPAICz possesses a more pronounced p‐type characteristic. With its optimized dipole moment, the DPAICz could show promise in enhanced hole extraction and improved transport across the interface.

We fabricated single‐junction PSCs with an inverted device architecture of ITO/NiO_x_/HSL/perovskite/LiF/C60/BCP/Ag to evaluate the photovoltaic performance of applying DPAICz as HSL in PSCs referenced to 2PACz. To achieve better device performance, a thin inorganic NiO_x_ layer was introduced between the ITO and HSL in both single‐junction devices and tandems. The best‐performing 2PACz‐based perovskite solar cells with a bandgap of 1.68 eV achieved a PCE of 21.74%, with an open‐circuit voltage (*V*_OC_) of 1.22 V, a short‐circuit current density (*J*_SC_) of 21.0 mA cm⁻^2^, and a fill factor (FF) of 84.89%. In comparison, devices using DPAICz as the HSL exhibited significantly enhanced performance, achieving a champion PCE of 23.42%, a *V*_OC_ of 1.25 V, a *J*_SC_ of 21.62 mA cm⁻^2^, and an FF of 86.67% (Figure 3d and Table S3, Supporting Information). To verify the reliability of the data, the external quantum efficiency (EQE) of the device was measured to calibrate the *J*_SC_ (Figure S20, Supporting Information). The integrated photocurrent of the champion device (21.14 mA cm⁻^2^) aligns closely with the value derived from the *J‐V* measurements. We attributed the enhanced photovoltaic performance in DPAICz‐based devices to its superior hole extraction efficiency.

We measured the steady‐state photoluminescence (PL) and time‐resolved PL (TRPL) spectra of perovskite film deposited on HSL‐modified ITO to estimate the difference in hole extraction ability between 2PACz and DPAICz. It is noted that the PL intensity of perovskite film deposited on top of DPAICz‐modified ITO is weaker (Figure S21, Supporting Information), suggesting the higher hole extraction efficiency of DPAICz.^[^ ^30^ ^]^ To mitigate the influence of charge accumulation at the interface under high laser fluence, we measured the TRPL signals at both high and low laser fluences (Figure S22, Supporting Information). We derived the differential lifetime from the TRPL measurements to better illustrate the differences in interfacial carrier dynamics. As shown in the Figure S23 (Supporting Information), the first time interval of DPAICz, which is predominantly governed by charge transfer, was shorter than that of 2PACz. This trend was more pronounced at low laser fluence (Figure 3e), verifying the superior hole extraction efficiency for DPAICz compared to 2PACz. Under high laser fluence, the first time interval governed by charge transfer and the second time interval governed by carrier recombination were more distinct in DPAICz than 2PACz, further suggesting the reduced charge accumulation at the interface for DPAICz. The charge carrier extraction ability of the devices using 2PACz and DPAICz as HSL was also analyzed via transient photocurrent (TPC) measurements. The photocurrent decay profiles under short circuit condition (Figure S24, Supporting Information) showed that the decay time in devices with DPAICz decreased a lot compared to the reference devices with 2PACz, benefiting from the higher charge carrier extraction efficiency of DPAICz. Figure 3f shows a histogram of the PCE distribution for 15 devices, highlighting the excellent reproducibility of the PCE improvement achieved with DPAICz (refer to box charts for statistical photovoltaic parameters in Figure S25, Supporting Information).

We further investigated the long‐term operational stability of perovskite solar cells based on DPAICz in comparison to devices based on 2PACz. The thermal stability was evaluated by continuously heating the devices at 65 °C in accordance with the ISOS‐D‐2 protocol. The PCE of the 2PACz‐based devices dropped to 80% of their initial PCEs in less than 1200 h, whereas DPAICz‐based devices maintained over 98% of their initial PCEs after 1650 h (Figure S26, Supporting Information). The significant improvement in thermal stability can be attributed to the amorphous structure of DPAICz on the ITO and the dual‐anchoring groups, which lock the conjugated core at the interface in a split‐standing mode. The operational stability of PSCs under illumination was evaluated by aging the devices under continuous 1‐sun light soaking at 65 °C following the ISOS‐L‐2 protocol. The DPAICz‐based device retained over 95% of its maximum PCE after 1600 h, while the 2PACz‐based device dropped to 80% (Figure S27, Supporting Information). The storage stability of devices following the ISOS‐D‐1 protocol showed that the DPAICz‐based devices retained 98% of their initial PCEs after 5500 h, while the 2PACz‐based devices dropped to 80% after 2000 h (Figure S28, Supporting Information).

### 2.4 Photovoltaic Performances of Monolithic Silicon/perovskite Tandem Solar Cells

**Figure 4:**
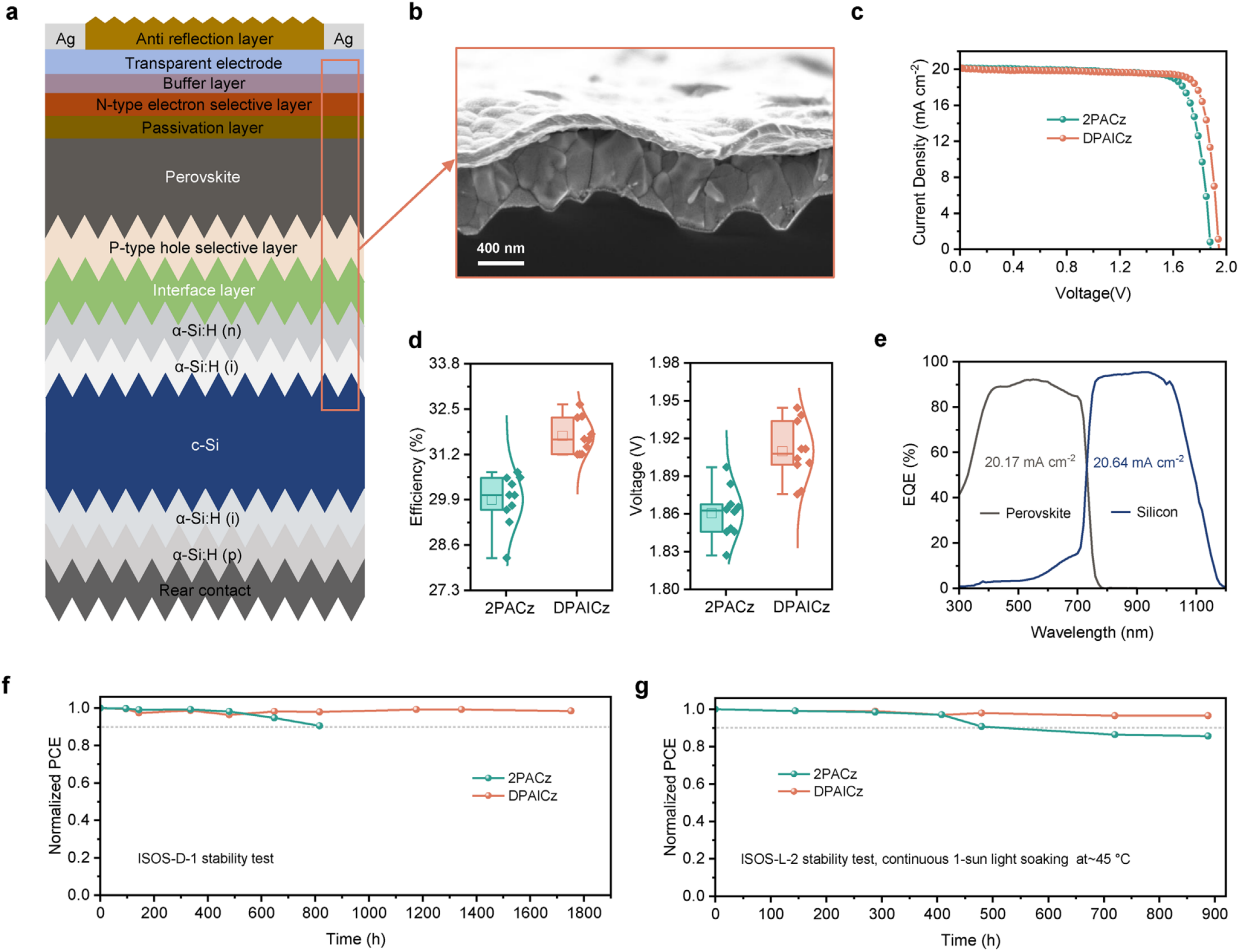
Photovoltaic performances and stability tests of monolithic silicon/perovskite tandem solar cells. a) Schematic of monolithic silicon/perovskite tandem solar cells built from a double‐side‐textured HJT cell. b) Scanning electron microscopy (SEM) cross‐section image of tandem. c) *J*‐*V* curves of monolithic silicon/perovskite tandem solar cells based on 2PACz and DPAICz. d) Statistics of PCE and *V*_OC_ values of monolithic silicon/perovskite tandem solar cells based on 2PACz and DPAICz. e) EQE of individual subcells for DPAICz‐based monolithic silicon/perovskite tandem solar cells. f) Evolution of the PCEs tracked following the ISOS‐D‐1 protocol. g) Evolution of the PCEs tracked under continuous 1‐sun light soaking at 45 °C following the ISOS‐L‐2 protocol.

Later, we incorporated DPAICz as the HSL in monolithic silicon/perovskite tandem solar cells, with 2PACz investigated in parallel for comparison. We used the commercialized heterojunction c‐Si subcell with silicon wafers featuring a mildly textured front surface and a heavily textured rear side. The full device architecture of the tandem devices employed in this study was illustrated in **Figure** **4**a and the corresponding scanning electron microscopy (SEM) cross‐section image was shown in Figure 4b (the fabrication process of the entire stack detailed in the methods).

Using DPAICz as hole selective layer for perovskite top cells, the best‐performing monolithic silicon/perovskite tandem solar cells achieved a PCE of 32.55%, a *V*oc of 1.94 V, a *J*sc of 20.15 mA cm⁻^2^, and an FF of 83.26%, compared with a PCE of 30.47% (*V*oc, ≈1.88 V; *J*sc, ≈20.14 mA cm^−2^; FF, ≈80.48%) for the champion control 2PACz‐based device (Figure 4c and Table S4, Supporting Information). The remarkable reproducibility of the PCE improvement in monolithic silicon/perovskite tandem solar cells achieved with DPAICz is evidenced by the statistical photovoltaic parameters shown in Figure 4d and Figure S29 (Supporting Information). The EQE measurements yielded integrated *J*sc values of 20.17 and 20.64 mA cm^−2^ (under AM 1.5G spectrum) for the perovskite and c‐Si subcells, respectively (Figure 4e), indicating good current matching. For the stability of DPAICz on textured silicon/ITO substrate under external stimuli, we tracked the performance of monolithic silicon/perovskite tandem solar cells based on DPAICz in comparison to device based on 2PACz. Figure 4f shows the evolution of the PCEs tracked following the ISOS‐D‐1 protocol. The PCE of the 2PACz‐based device dropped to 90% of its initial PCE in less than 810 h, whereas DPAICz‐based device maintained over 98% of its initial PCE after 1700 h. We also assessed the operational stability of device under illumination by aging them under continuous 1‐sun light soaking at 45 °C under open‐circuit condition, in accordance with the ISOS‐L‐2 protocol. As shown in Figure 4g, the control device dropped to 90% of its initial PCE after only 480 h, while the DPAICz‐based device maintained 96% of its initial PCE after 880 h. Moreover, the steady‐state power output at the maximum power point (MPP) of the monolithic textured silicon/perovskite tandem solar cells based on DPAICz over 600 s under ambient air conditions at room temperature was conducted to evidence that DPAICz‐based device exhibited stable current output, reaching a steady‐state efficiency of 32.13% (Figure S30, Supporting Information). Benefiting from its strong anchoring stability, the operational stability of DPAICz‐based monolithic silicon/perovskite tandem solar cells significantly outperformed that of devices with 2PACz, highlighting the critical role of DPAICz in achieving long‐term stable textured silicon/perovskite tandem solar cells.

## 3 Conclusion

We report a bipedal indolocarbazole‐based HSL material with excellent hole extraction efficiency and enhanced stability, which significantly improves the reliability of HSL on high‐roughness textured silicon surface, thereby enhancing both the efficiency and stability of monolithic silicon/perovskite tandem solar cells utilizing small‐molecule HSL. The π‐expanded conjugated core and split‐standing molecular configuration of DPAICz optimize its dipole moment, enhancing the hole extraction performance. Additionally, the incorporation of multiple anchoring groups facilitates the formation of bidentate anchors through two phosphonic acid groups with ITO, reinforcing its adhesion under external stimuli. This approach addresses the issues of dipole moment misalignment and weak stability on substrates, which are commonly encountered with traditional HSL materials such as 2PACz. Our study provides theoretical guidance for the design of efficient and stable HSL materials on textured silicon surface, advancing the commercialization of perovskite‐silicon tandem technology.

## Conflict of Interest

The authors declare no conflict of interest.

## Supporting information



Supporting Information

## Data Availability

The data that support the findings of this study are available from the corresponding author upon reasonable request.
