# Supplementary material for: Split‐Standing Molecular Engineering for Textured Silicon/Perovskite Tandems
Source: Adv Sci (Weinh). 2025 Jun 25;12(35):e05288. doi: 10.1002/advs.202505288 (PMC12462990; doi:10.1002/advs.202505288)
Supplement: Supplementary file 1 — Supporting Information [file ADVS-12-e05288-s001.docx]

**Supporting Information**

Split-Standing Molecular Engineering for Textured Silicon/Perovskite Tandems

Xiaonan Wang,^†[a,b]^ Yuan Tian,^†[a,b]^ Libing Yao,^[b]^ Shaochen Zhang,^[a,b]^ Qingqing Liu,^[b]^ Ke Zhao,^[a,b]^ Jiazhe Xu,^[a,b]^ Jingjing Zhou,^[a,b]^ Caner Deger,^[c]^ Ilhan Yavuz,^[c]^ Jingjing Xue,^[a]^ and Rui Wang*^[b]^

[a] X. Wang, Y. Tian, S. Zhang, K. Zhao, J. Xu, J. Zhou, Prof. J. Xue
State Key Laboratory of Silicon and Advanced Semiconductor Materials, School of Materials Science and Engineering
Zhejiang University
Hangzhou 310027, China

[b] X. Wang, Y. Tian, Dr. L. Yao, S. Zhang, Dr. Q. Liu, K. Zhao, J. Xu, J. Zhou, Prof. R. Wang
Department of Materials Science and Engineering, School of Engineering
Westlake University
Hangzhou 310024, China
E-mail: wangrui@westlake.edu.cn

[c] Prof. C. Deger, Prof. I. Yavuz
Physics Department
Marmara University
Kadikoy 34722, Istanbul, Turkiye

^†^ These authors contributed equally to this work.

**Methods:**

**Materials:**

Unless otherwise stated, all materials were commercially available and used without any further purification. Compound 11,12-Dihydroindolo[2,3-a]carbazole, 1,2-dibromoethane, tetrabutylammonium bromide (TBABr), triethyl phosphite (98%), methanol (99.7%), 1,4-dioxane (99.7%, SafeDry, with molecular sieves) were purchased from Innochem. Bromotrimethylsilane (TMSBr, 98%), Chloroform-d (CDCl_3_, 99.8%) and dimethyl sulfoxide-d6 (DMSO-*d_6_*, 99%) were purchased from J&K Scientific. Potassium hydroxide (KOH, 85%) was purchased from Greagent. The transparent ITO glass substrates (10 Ω per square, transmittance 88%) was purchased from Shenzhen Huayu United Technology Co., Ltd. NiO_x_ nanoparticle powder, Lead iodide (PbI_2_, purity 99.999%), methylammonium bromide (MABr, purity 99.9%), formamidinium iodide (FAI, purity 99.99%), methylammonium chloride (MACl, purity 99.9%), lithium fluoride (LiF, purity 99.99%) and C_60_ (purity 99.9%) were all purchased from Advanced Election Technology Co., Ltd. Ethylenediammonium diiodide (EDADI_2_), Bathocuproine (BCP, purity 99%), cesium iodide (CsI, purity 99.999%), (2-(9H-carbazol-9-yl)ethyl)phosphonic acid (2PACz, purity 99%) were purchased from Xi’an Polymer Light Technology Corp. Inc. N, N-Dimethylformamide (DMF, extra dry, purity 99.8%), dimethyl sulfoxide (DMSO, anhydrous, purity 99.9%), isopropanol (IPA, extra dry, purity 99.5%), methanol (extra dry, purity 99.8%), chlorobenzene (CB, anhydrous, purity 99.9%), ethyl acetate (EA, extra dry, purity 99.5%) and silver (Ag, purity 99.99%) were purchased from Sigma-Aldrich Inc.

**Single-junction wide-bandgap perovskite solar cells fabrication**

The pre-patterned ITO glass substrate was washed consecutively with detergent, deionized water, acetone, and isopropanol (IPA), each under 30 minutes of sonication. The clean ITO glass substrate was treated with ultraviolet-ozone for 35 min. Then the substrate was spin-coated with a thin layer of NiO_x_ nanoparticle film (10 mg/ml NiO_x_ water solution) at 3000 rpm for 30 s, and annealed in ambient air at 120 °C for 20 min and then transferred into glovebox filled with nitrogen. 2PACz (0.6 mg mL^-1^) or DPAICz (0.6 mg mL^-1^) in mixed solvent of methanol and DMF at volume ratio of 3:1 was spin-coated at 1,000 rpm for 10 s and 3,000 rpm for 40 s, followed by annealing at 100 °C for 30 min. For the preparation of perovskite films with a composition FA_0.74_Cs_0.26_PbI_2.43_Br_0.43_Cl_0.14_, 1.65 M perovskite precursor solution was prepared by dissolving CsI, FAI, MACl, PbCl_2_, CsCl, PbBr_2_ and PbI_2_ in 1 mL mixed solvent of DMF and DMSO at volume ratio 4:1 and the solution was stirred overnight. The filtered perovskite precursor was then spin-coated on substrate at 5000 rpm for 50 s in nitrogen glovebox. 200 µL of CB anti-solvent was dropped quickly at 25 s during the spinning step. Afterwards, the film was annealed at 110 °C for 30 min. The IPA solution of EDADI_2_ (0.3 mg/mL) was spin-coated on the perovskite surface at 5000 rpm for 30 s as a passivation layer and then was annealed at 110 °C for 10 min. Then, LiF (0.5 nm) at a rate of 0.1 Å s^-1^, C_60_ (40 nm) at a rate of 0.2 Å s^-1^ and BCP (6 nm) at a rate of 0.2 Å s^-1^ were thermally evaporated on the perovskite thin film sequentially in a vacuum chamber (<5.0×10^-6^ Pa). Finally, silver electrode (120 nm) at a rate of 1.5 Å s^-1^ was deposited by thermal evaporation in a vacuum chamber (<1.0×10^-5^ Pa). All of these processes were conducted in the N_2_ protected glove box with the contents of O_2_ and H_2_O less than 0.1 ppm.

**Monolithic silicon/perovskite tandem solar cells fabrication**

The 105 mm * 210 mm silicon wafers were laser-cut into 25 mm×25 mm pieces, with the ~1.1 cm² contacted areas of ITO positioned at the center and then were subjected to UV-Ozone treatment for 20 min before transferred into the glovebox. The hole transport layer was spin-coated onto the silicon/ITO substrates following the abovementioned method. After cooling down to room-temperature, perovskite film and the surface passivation layer were coated on these layers following the abovementioned method. After that, 0.5 nm LiF and 20 nm C_60_ were deposited by thermal evaporation. And then 20 nm SnO_2_ was then deposited by atomic layer deposition (ALD) using a Veeco system. The substrate temperature was maintained at 85 °C during ALD deposition with TDMASn precursor source at 60 °C. The pulse and purge time for Tetrakis(dimethylamino)tin(IV) (TDMASn) is 1 and 10 s with a 90 sccm carrier gas of nitrogen, for H_2_O is 0.15 and 10 s with 90 sccm N_2_. 180 cycles were used. 60 nm IZO was sputtered from a 3-inch IZO ceramic target on top of the SnO_2_ through a 1.1 cm^2^ shadow mask. Ag contact finger with a thickness of 400 nm was thermally evaporated using a high precision shadow mask. Finally, 100 nm LiF was thermally evaporated as an anti-reflection layer.

**Device Characterization**

The *J-V* characteristics of photovoltaic cells were measured using a Keithley 2400 source measure unit under a simulated AM 1.5G spectrum, with an Oriel 9600 solar simulator. The illumination light intensity (100 mW cm^-2^ AM 1.5G) was calibrated by a silicon reference cell. Typically, the single-junction devices were measured in reverse scan (1.20 V → 0 V, step 0.02 V) and the tandem devices were measured in reverse scan (2.00 V → 0 V, step 0.02 V). External quantum efficiencies (EQEs) were measured using an integrated system (Enlitech Technology) and a lock-in amplifier with a current preamplifier under short circuits’ condition. Transient photocurrent decay (TPC) measurements under the short-circuit condition were conducted by photo-electrochemical measurement system.

**Device stability tests**

The light-heat stability test following the ISOS-L-2 protocol was conducted in the nitrogen atmosphere with continuous heating at ~65 ℃ and under continuous AM 1.5G illumination (100 mW cm^-2^, white LED) at open-circuit condition for the unencapsulated devices. The thermal stability test following the ISOS-D-2 protocol was conducted in the dark and a nitrogen atmosphere with continuously heating the unencapsulated device at ~65 °C. The storage stability test following the ISOS-D-1 protocol was conducted in the dark and a nitrogen atmosphere at room temperature. The voltage at the MPP was automatically adjusted and applied, and the power output of the devices was tracked over time.

**Fabrication of 2PACz or DPAICz modified substrates rinsed by solvent**

For fresh HSL film fabrication, 2PACz and DPAICz were deposited onto substrates (Glass/ITO substrate or textured silicon/ITO substrate) following the same process used for the device fabrication (spin-coating at 1,000 rpm for 10 s and 3,000 rpm for 40 s, followed by annealing at 100 °C for 30 min). Subsequently, different volumes of ethanol (50 µl, 150 µl, 250 µl) were applied to rinse the HSL films deposited on the substrate by a dynamic spin-coating process at 3000 rpm for 30 s. Finally, the substrates were annealed at 100 °C for 10 min to remove residual ethanol solvent. To eliminate potential effects of the additional 10 min annealing step on the HSL films, freshly deposited HSL films without solvent rinsing treatment were also subjected to an identical annealing treatment at 100 °C for 10 min.

**Materials characterizations**

**KPFM and c-AFM.** For the KPFM and c-AFM measurements of the 2PACz and DPAICz fresh films, the films were prepared on cleaned and dried ITO glass substrates in a nitrogen glovebox by spin-coating 2PACz and DPAICz solutions (0.6 mg mL^-1^ in mixed solvent of methanol and DMF at volume ratio of 3:1) at 1,000 rpm for 10 s and 3,000 rpm for 40 s, followed by annealing at 100 °C for 30 min. The samples were then transferred into a container filled with nitrogen after cooling down to the room temperature. The as-prepared samples were subsequently sent out for KPFM and c-AFM measurements, which were performed with a Jupiter XR Oxford Instruments. For all c-AFM measurements, a bias of 600 mV was applied.

**Contact angle measurements.** For the contact angle measurements, the 2PACz and DPAICz films were prepared on cleaned and dried Glass/ITO substrates or textured silicon/ITO substrates using the same method as for the AFM measurements. The 2PACz or DPAICz deposited on different substrates were recorded in a range of 0-180° with high precision (±0.1° accuracy) using a CA200 contact angle analyser.

**Photoelectron spectroscopy.** For the high-resolution X-ray photoelectron spectroscopy (HR-XPS) and ultra-violet photoelectron spectroscopy (UPS) measurements, the 2PACz and DPAICz films were prepared using the same method as described above for the AFM measurements. The samples were then loaded into the X-ray photoelectron spectrometer (ThermoFisher ESCALAB Xi+) and an Al Kα X-ray was used as the excitation source. The UPS measurements were carried out to determine the work function and the position of valence band maximum of materials. A helium discharge lamp, emitting ultraviolet energy at 21.2 eV, was used for excitation. All UPS measurements were performed using standard procedures with a -10 V bias applied between the samples and detectors. Clean and etched gold was used as a reference.

**Fourier transform infrared spectroscopy.** The 2PACz and DPAICz powders were stored in brown vials filled with nitrogen before being sent out for measurement. To measure the combination between the HSL materials and ITO, 2PACz and DPAICz powders were mixed with indium oxide powder in stoichiometric ratio, respectively, and then dissolved in DMF solution. The mixture was stirred at 110 °C for 20 minutes. The solutions were deposited onto glass substrates and dried at 130 °C in a nitrogen glovebox to remove the solvent. The dried powder was scraped off and stored in brown vials filled with nitrogen before being sent out for measurement. The attenuated total reflection (ATR) spectra were collected at room temperature by Thermo Fischer Scientific Nicolet IS50 FT-IR spectrometer. Spectral resolution was set to 1 cm^-1^, aperture to 4 mm, and spectra were acquired by averaging 64 scans.

**PL and TRPL spectroscopy.** For photoluminescence (PL) and time-resolved photoluminescence (TRPL) measurements, the perovskite layers were prepared according to the methods described above. The PL signal was acquired through a time-correlated single-photon-counting StrobeLock system with the excitation wavelength at 490 nm. The total instrument response function for the PL decay was <200 ps, and the temporal resolution was <30 ps. The TRPL spectra were measured by a steady-state transient fluorescence spectrometer (FLS1000). A picosecond laser diode with a wavelength of 405 nm was used as the excitation source. The energy density of laser for TRPL measurements under different laser influence was set to be 2.6 nJ cm^-2^ and 26.2 nJ cm^-2^ respectively.

**XRD.** For the XRD measurements, 2PACz and DPAICz films were prepared by spin-coating the 2PACz and DPAICz solutions with a higher concentration of 0.02 mmol ml^-1^ in the mixed solvent indicated previously to make thicker films for better signals. Afterwards, the film was annealed at 100 °C for 30 min as fresh sample. XRD data were collected in reflection mode at room temperature on a D8 Advance diffractometer equipped with a 1D LynxEye detector using monochromated Cu-Kα radiation.

**Scanning electron microscopy.** The surface and cross-sectional morphologies of the perovskite films were determined using a field emission environment scanning electron microscope (QuattroS). The perovskite films were prepared using the same method as mentioned earlier.

**First-principles calculations**

The geometrical optimization and electronic properties of the molecules were conducted by Gaussian 09 with B3LYP as the exchange-correlation function and 6-311G (d, p) basis set. The binding energies were calculated by Vienna ab initio simulation package (VASP) software. The plane-wave basis set and the projected augmented wave (PAW) method were utilized in this work. The Perdew-Burke-Ernzerhof (PBE) functional, which applies the generalized-gradient approximation for the exchange-correlation functional, was used for both self-consistent field calculations and geometry optimization. Geometry optimizations were performed with a 4 × 4 × 1 Γ-centered k-point mesh and a plane wave energy cutoff of 400 eV. The atomic positions and cell volumes were relaxed using the conjugate gradient algorithm until the residual forces on all atoms were below 0.02 eV/Å. Furthermore, a vacuum layer of 10-15 Å was introduced along the z direction to separate the periodic slab-molecule structure. The binding energies of A and B were calculated as follows: 𝐸_𝑏𝑖𝑛𝑑_ = 𝐸_𝐴𝐵_ − 𝐸_𝐴_ − 𝐸_𝐵_.

**Synthesis and characterization of DPAICz**

The detailed synthetic route and molecular characterizations of DPAICz are shown in Scheme S1 and Figure S31-S38.


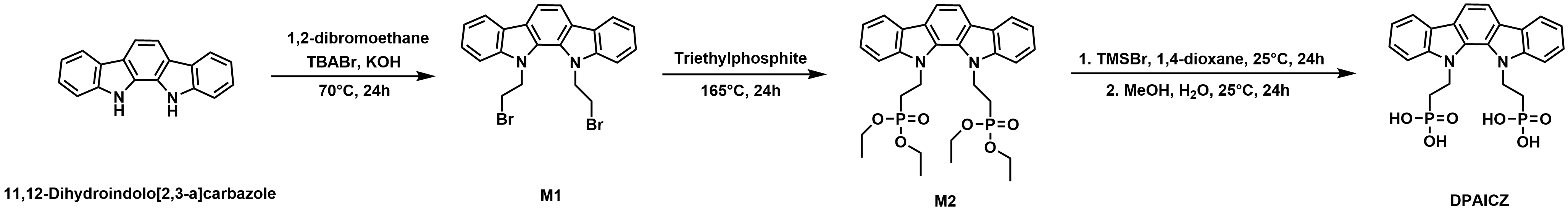
^1^H NMR, ^13^C NMR and ^31^P NMR spectra were measured on Bruker AVANCE NEO 600 MHz spectrometers.

**Scheme S1.** Synthetic route of DPAICz.

**M1** Under argon atmosphere, 11,12-Dihydroindolo[2,3-a]carbazole (1.54 g, 6 mmol) was dissolved in 1,2-dibromoethane (16.0 eq, 18.03 g, 8.27 mL, 96 mmol), tetrabutylammonium bromide (0.1 eq, 193 mg, 0.6 mmol) and 50% KOH aqueous solution (5.0 eq) were added subsequently. The mixture was stirred at 70 °C overnight. After completion of the reaction, extraction was done with ethyl acetate. The combined organic layer was dried over anhydrous Na_2_SO_4_ and the solvent was distilled off under reduced pressure. The crude product was purified by column chromatography (n-hexane: ethyl acetate 10:1 *v*:*v*) to give M1 (985 mg, 35%) of white powder.

^1^H NMR (600 MHz, Chloroform-d) δ 8.048 (d, J = 7.7 Hz, 2H), 7.895 (s, 2H), 7.505 (d, J = 8.1 Hz, 2H), 7.441 (t, J = 7.0 Hz, 2H), 7.280 (t, J = 7.4 Hz, 2H), 4.859 – 4.809 (m, 4H), 3.307 – 3.261 (m, 4H).

^13^C NMR (151 MHz, Chloroform-d) δ 141.880, 128.093, 125.529, 124.908, 124.315, 120.307, 119.246, 112.919, 110.232 48.700 26.423.

**M2** Under argon atmosphere, M1 (940 mg, 2 mmol) was dissolved in triethyl phosphite (20.0 eq, 6.7 g, 6.8 mL, 40.0 mmol) in a Schlenk flask. The reaction was stirred at 165 °C overnight. After completion of the reaction, the solvent was distilled off under reduced pressure. The crude product was purified by column chromatography (n-hexane: acetone 1:5 *v*:*v*) to give M2 (994 mg, 85%) of colorless oil.

^1^H NMR (600 MHz, Chloroform-d) δ 8.102 (d, J = 7.6 Hz, 2H), 7.941 (s, 2H), 7.563 (d, J = 8.1 Hz, 2H), 7.493 (t, J = 7.6 Hz, 2H), 7.329 (t, J = 7.4 Hz, 2H), 4.856 (dd, J = 17.2, 5.5 Hz, 4H), 4.006 – 3.942 (m, 8H), 1.878 – 1.814 (m, 4H), 1.235 (t, J = 7.1 Hz, 12H).

^13^C NMR (151 MHz, Chloroform-d) δ 143.346, 129.703, 127.088, 125.798, 121.145, 120.148, 113.808, 111.844, 61.824, 42.857, 24.869, 23.965, 16.332.

^31^P NMR (243 MHz, Chloroform-d) δ 27.451.

**DPAICz** Under argon atmosphere, M2 (584 mg, 1 mmol) was dissolved in dry 1,4-dioxane (10 mL) in a Schlenk flask and bromotrimethylsilane (10.0 eq, 1.5 g, 10.0 mmol) was added dropwise. The mixture was stirred for 24 h at room temperature. Afterwards, the solvent was distilled off under reduced pressure and the residue was dissolved in methanol (5 mL). Next, distilled water was added dropwise until the solution became opaque. The product was filtered off and washed with water to give DPAICz (425 mg, 90%) as white powder.

^1^H NMR (600 MHz, DMSO-d6) δ 8.223 (d, J = 7.6 Hz, 2H), 8.051 (s, 2H), 7.685 (d, J = 8.1 Hz, 2H), 7.510 (t, J = 7.6 Hz, 2H), 7.310 (t, J = 7.4 Hz, 2H), 4.877 – 4.784 (m, 4H), 1.845 (dt, J = 17.8, 8.4 Hz, 4H).

^13^C NMR (151 MHz, DMSO-d6) δ 142.770, 129.168, 126.141, 124.756, 121.025, 120.474, 113.896, 112.059, 43.539, 28.127, 27.273.

^31^P NMR (243 MHz, DMSO-d6) δ 20.724.

**
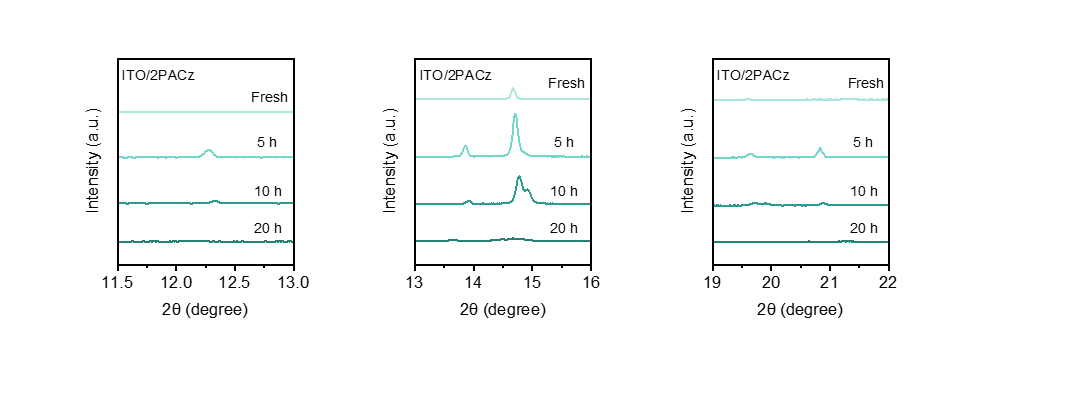
**

**Figure S1.** Details of XRD patterns of 2PACz on ITO during 20 hours at 150 °C heating conditions.





**Figure S2.** The XPS spectra of Sn 3d core level for 2PACz-modified Glass/ITO substrate rinsed by different volume of ethanol.





**Figure S3.** The XPS spectra of In 3d core level for 2PACz-modified textured silicon/ITO substrate rinsed by different volume of ethanol.


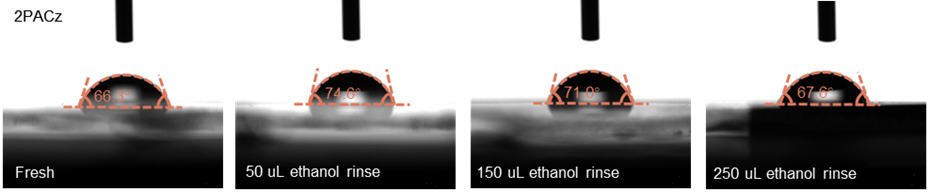


**Figure S4.** Contact angle of water for 2PACz deposited on Glass/ITO substrate after rinsing with different volume of ethanol.


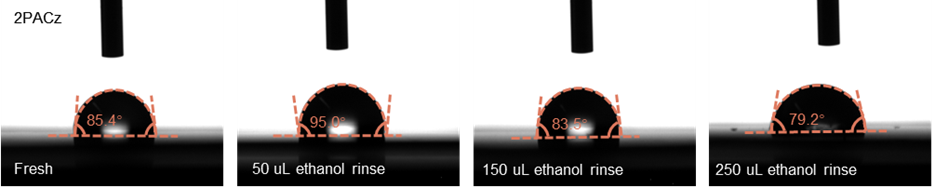


**Figure S5.** Contact angle of water for 2PACz deposited on textured silicon/ITO substrate after rinsing with different volume of ethanol.


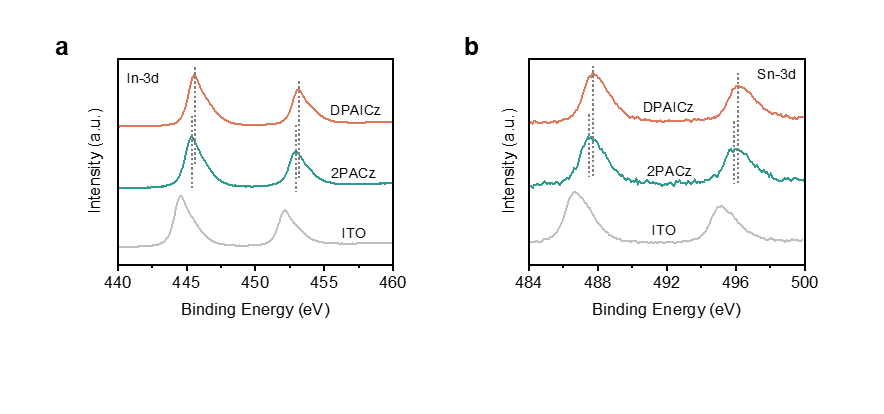


**Figure S6.** The XPS spectra of In 3d core level and Sn 3d core level for bare ITO, 2PACz and DPAICz modified ITO.


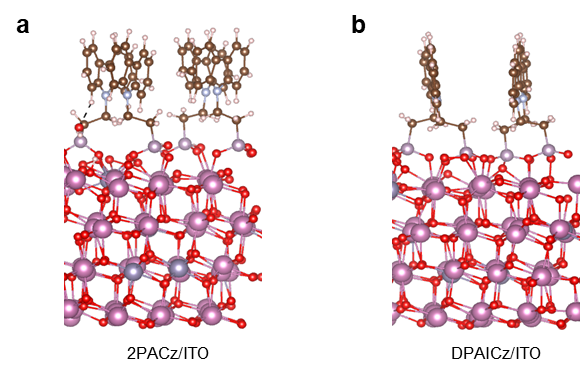


**Figure S7.** Theoretical models of 2PACz and DPAICz assembled on the ITO surface.


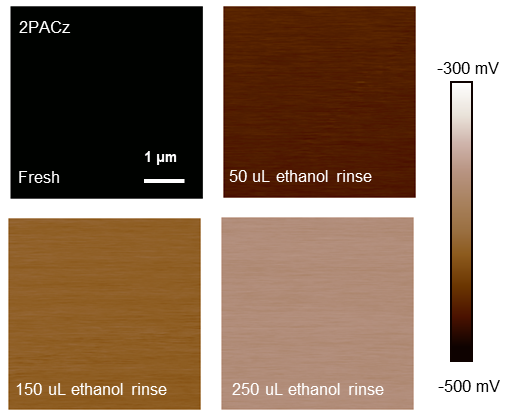


**Figure S8.** Surface potential images obtained by scanning Kelvin probe force microscopy (KPFM) after rinsing the 2PACz films deposited on Glass/ITO substrate with different volume of ethanol.


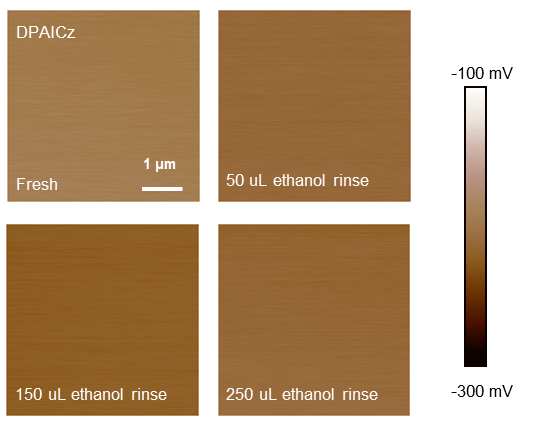


**Figure S9.** Surface potential images obtained by scanning Kelvin probe force microscopy (KPFM) after rinsing the DPAICz films deposited on Glass/ITO substrate with different volume of ethanol.





**Figure S10.** Statistical surface potential signals of the 2PACz films and DPAICz films deposited on Glass/ITO substrate after rinsing by different volume of ethanol.


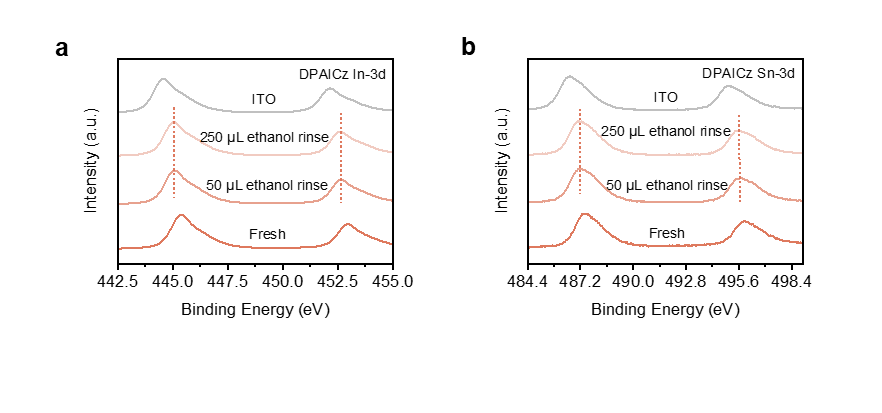


**Figure S11.** The XPS spectra of In 3d core level and Sn 3d core level for DPAICz deposited on Glass/ITO substrate after rinsing by different volume of ethanol.


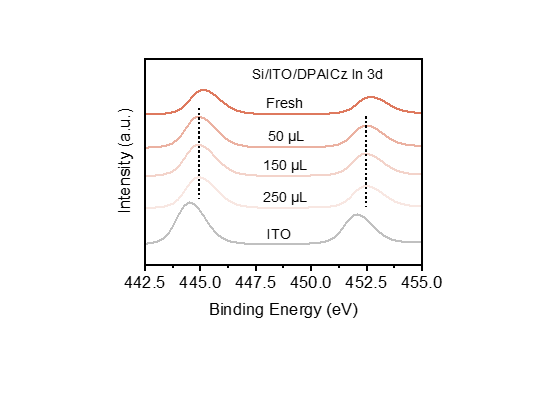


**Figure S12.** The XPS spectra of In 3d core level for DPAICz deposited on textured silicon/ITO substrate rinsed by increasing volume of ethanol.


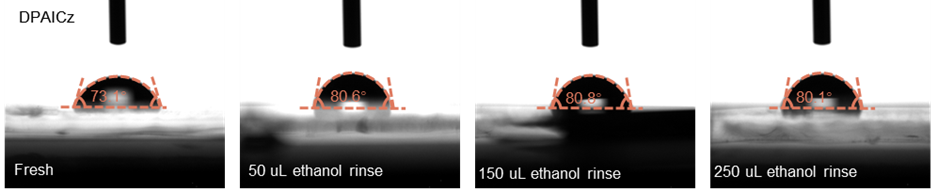


**Figure S13.** Contact angle of water for DPAICz deposited on Glass/ITO substrate after rinsing with different volume of ethanol.


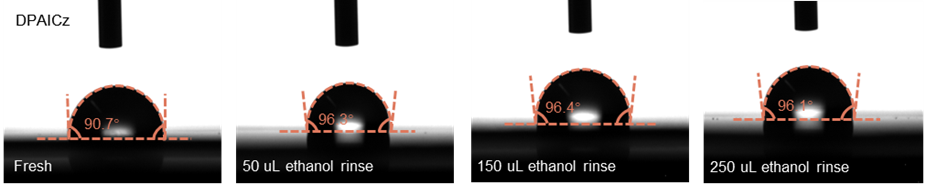


**Figure S14.** Contact angle of water for DPAICz deposited on silicon/ITO substrate after rinsing with different volume of ethanol.


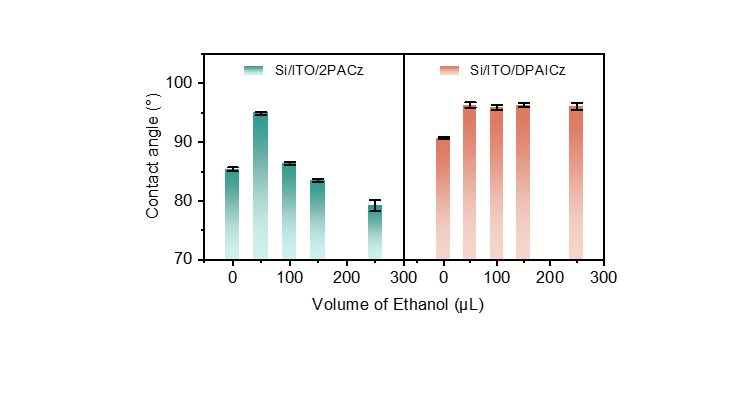


**Figure S15.** Statistical contact angle of the 2PACz films and DPAICz films deposited on silicon/ITO substrate after rinsing by different volume of ethanol.


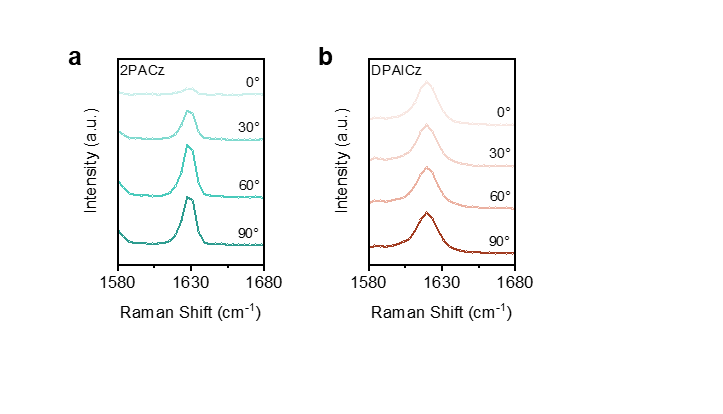


**Figure S16.** Polarization-dependent Raman spectroscopy of the 2PACz (a) and DPAICz (b) films.


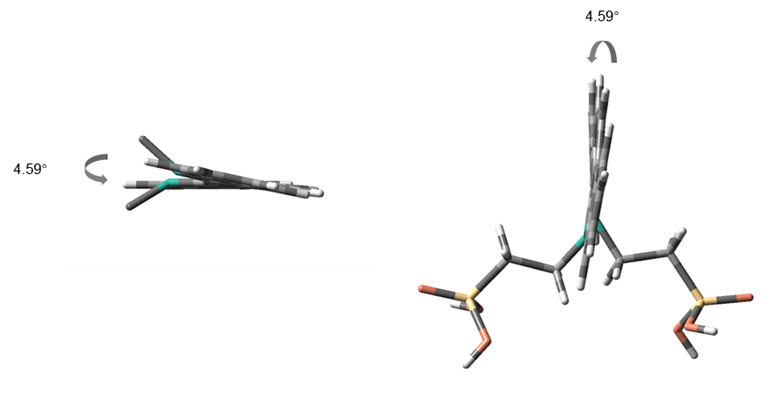


**Figure S17.** The dihedral angles of the π-scaffolds of DPAICz. The dihedral angles were measured through the plane defined by the central benzene ring and the lateral indole nucleus on either side.


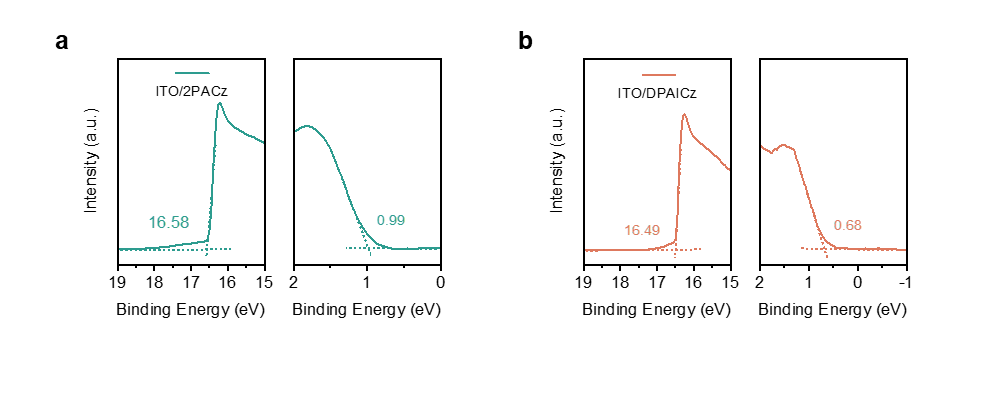


**Figure S18.** UPS results of the ITO modified with different molecules.


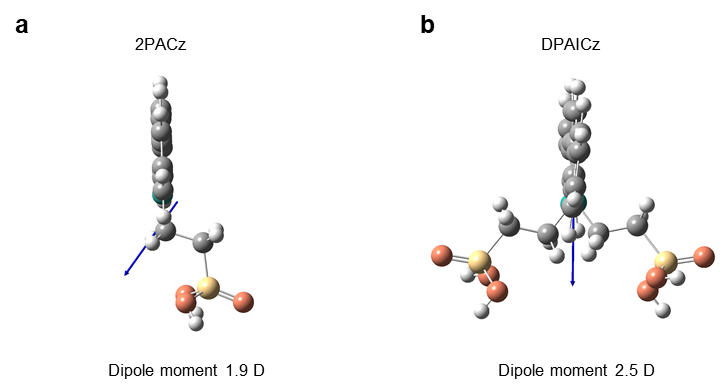


**Figure S19.** Dipole moment of 2PACz and DPAICz.


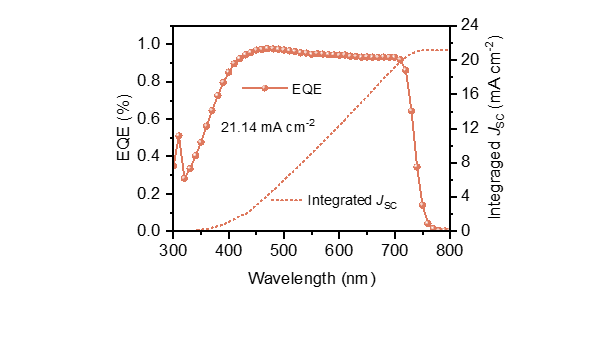


**Figure S20.** EQE of the single-junction wide-bandgap perovskite solar cells (1.68 eV) fabricated with DPAICz.


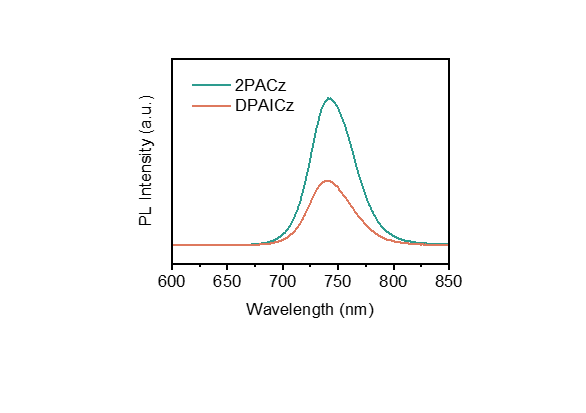


**Figure S21.** PL emission of wide band-gap perovskite films deposited on 2PACz and DPAICz.


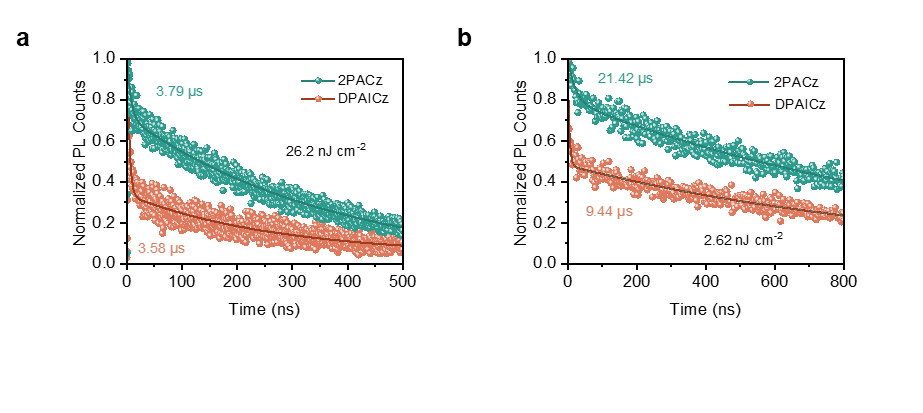


**Figure S22.** The TRPL spectra at different laser fluence for perovskite films deposited on 2PACz and DPAICz.


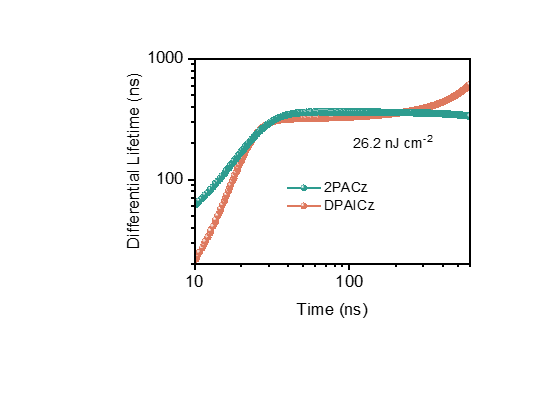


**Figure S23.** The differential lifetime derived from TRPL measurements at high laser fluence of 26.2 nJ cm^-2^ for perovskite films in contact with 2PACz and DPAICz.


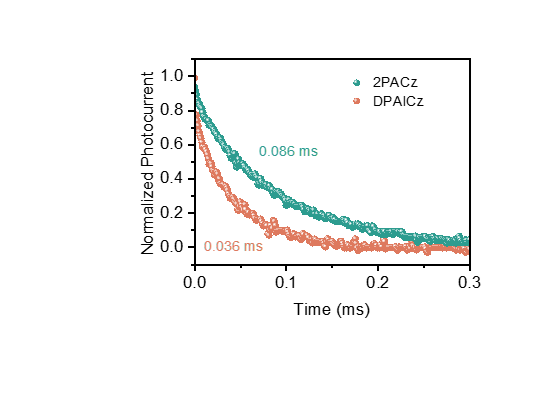


**Figure S24.** Transient photocurrent decay of devices with two HSL.


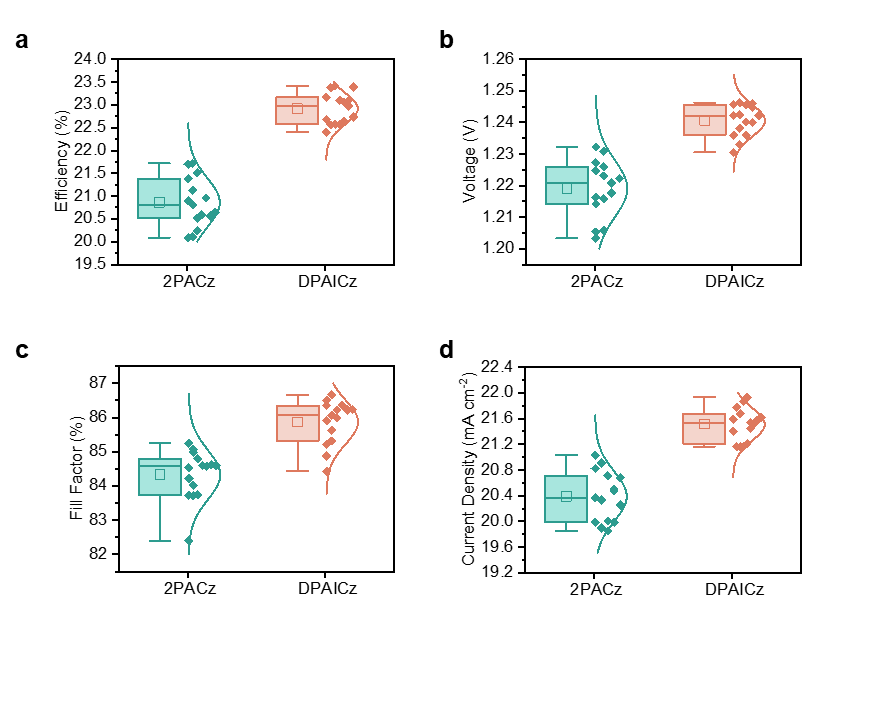


**Figure S25.** Statistics of photovoltaic parameters (PCE, *V*_OC_, FF and *J*_SC_) obtained from the *J*-*V* characteristics of the single-junction wide-bandgap perovskite solar cells (1.68 eV) based on 15 devices for each condition.


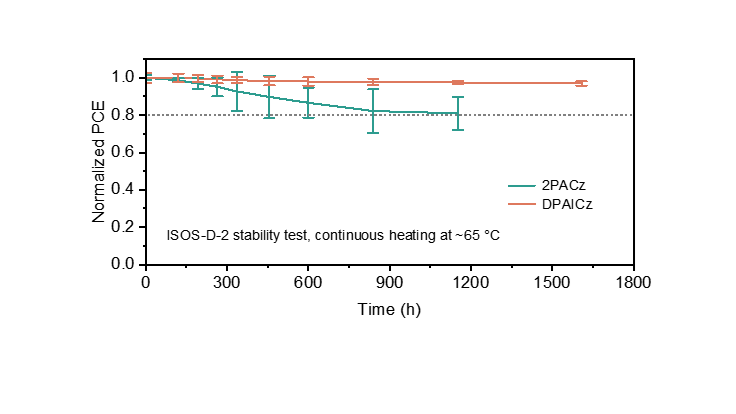


**Figure S26.** Evolution of the PCEs tracked under continuous heating at ~65 °C following the ISOS-D-2 protocol. Error bars represent the standard deviation of five devices for each condition.


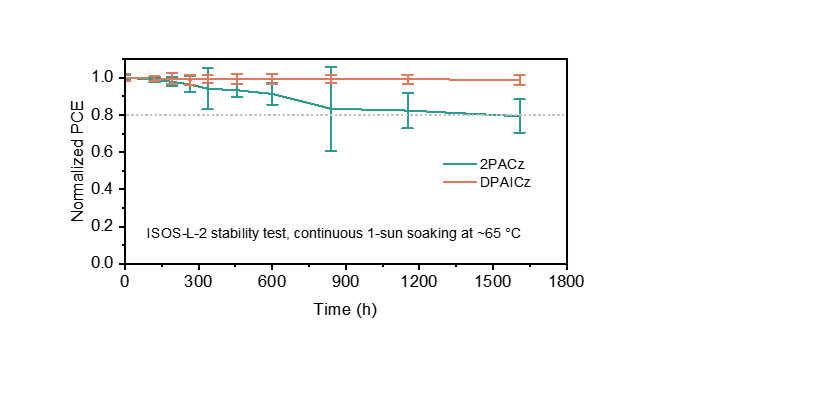


**Figure S27.** Evolution of the PCEs tracked under continuous 1-sun light soaking at ~65 °C following the ISOS-L-2 protocol. Error bars represent the standard deviation of five devices for each condition.


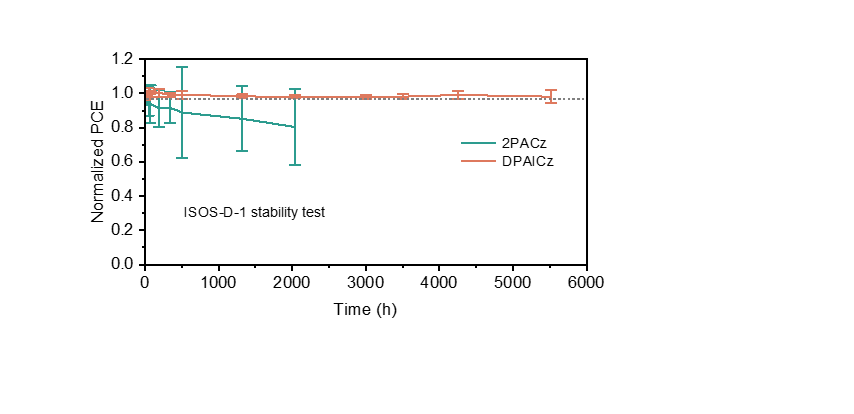


**Figure S28.** Evolution of the PCEs tracked following the ISOS-D-1 protocol. Error bars represent the standard deviation of five devices for each condition.


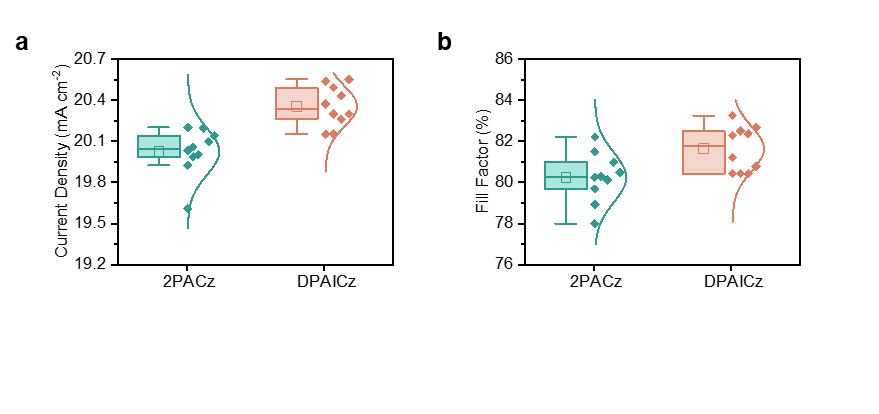


**Figure S29.** Statistics of photovoltaic parameters (FF and *J*_SC_) obtained from the *J*-*V* characteristics of monolithic perovskite/silicon tandem solar cells based on 10 devices for each condition.


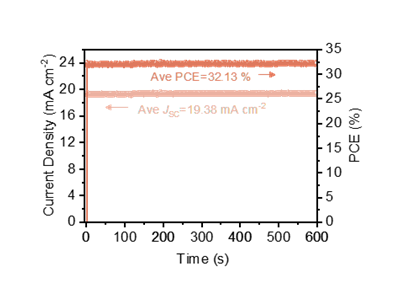


**Figure S30.** Steady-state power output at the maximum power point (MPP) of the monolithic perovskite/silicon tandem solar cells based on DPAICz.


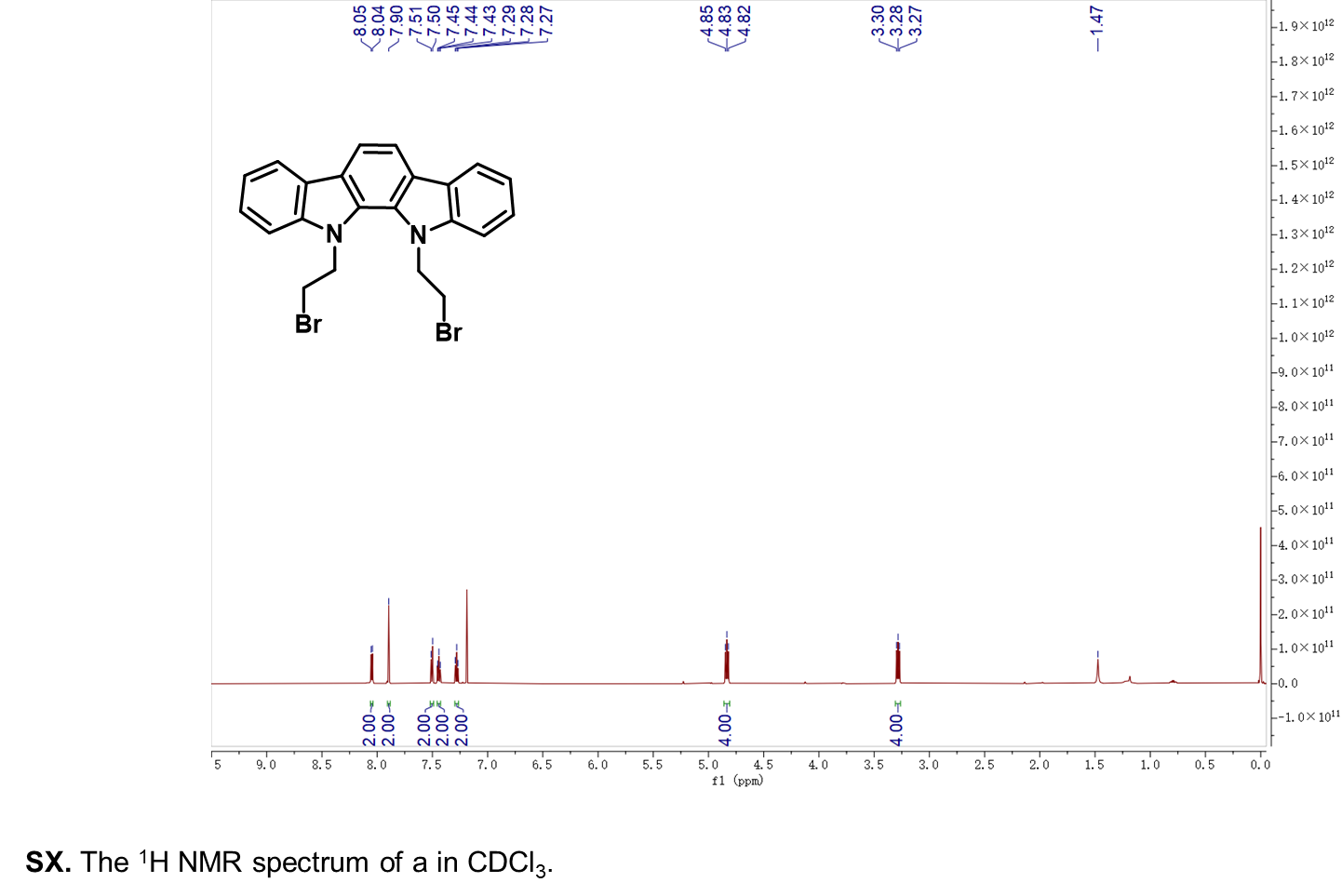


**Figure S31.** The ^1^H NMR spectrum of M1 in CDCl_3_.


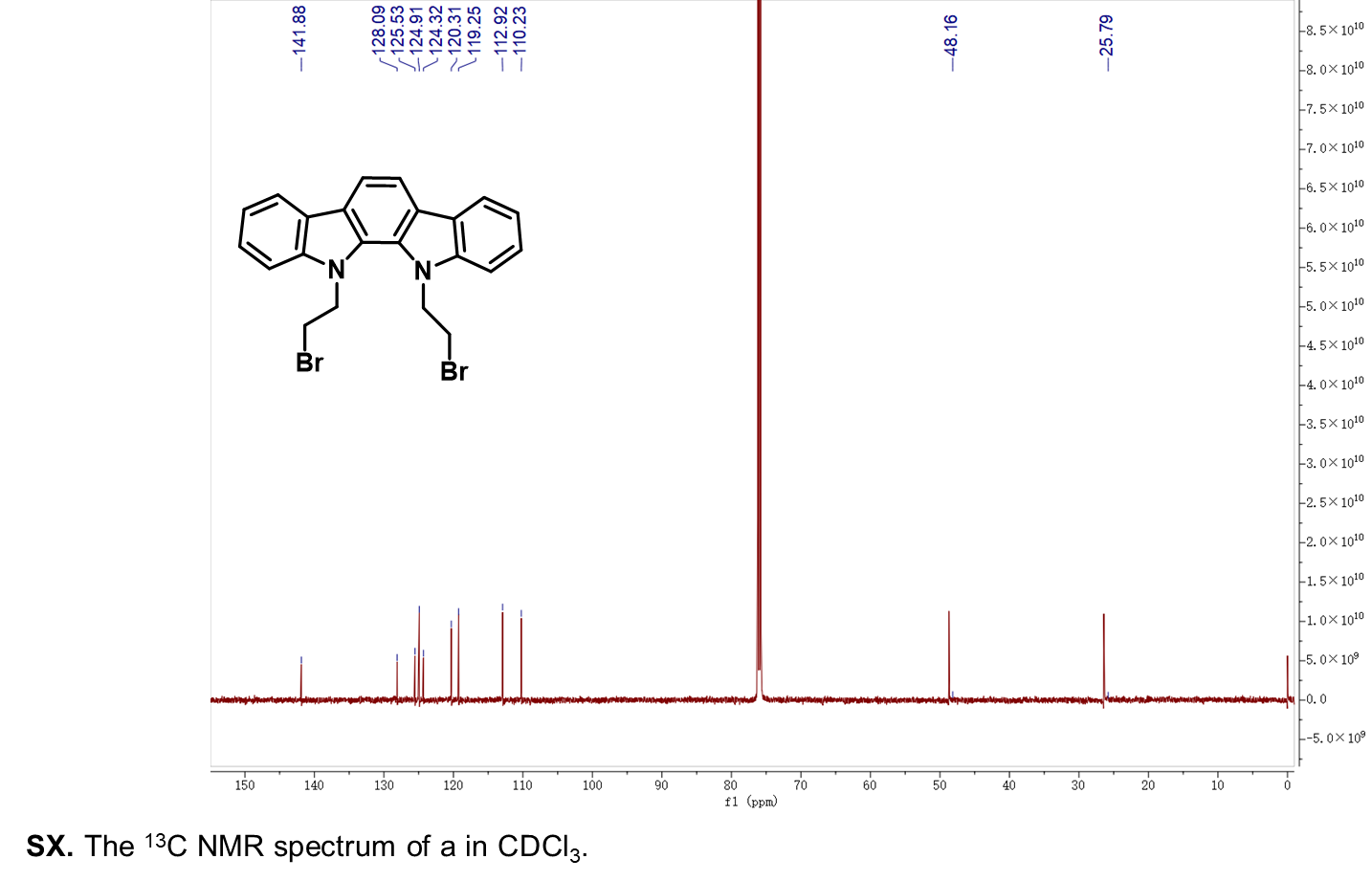


**Figure S32.** The ^13^C NMR spectrum of M1 in CDCl_3_.


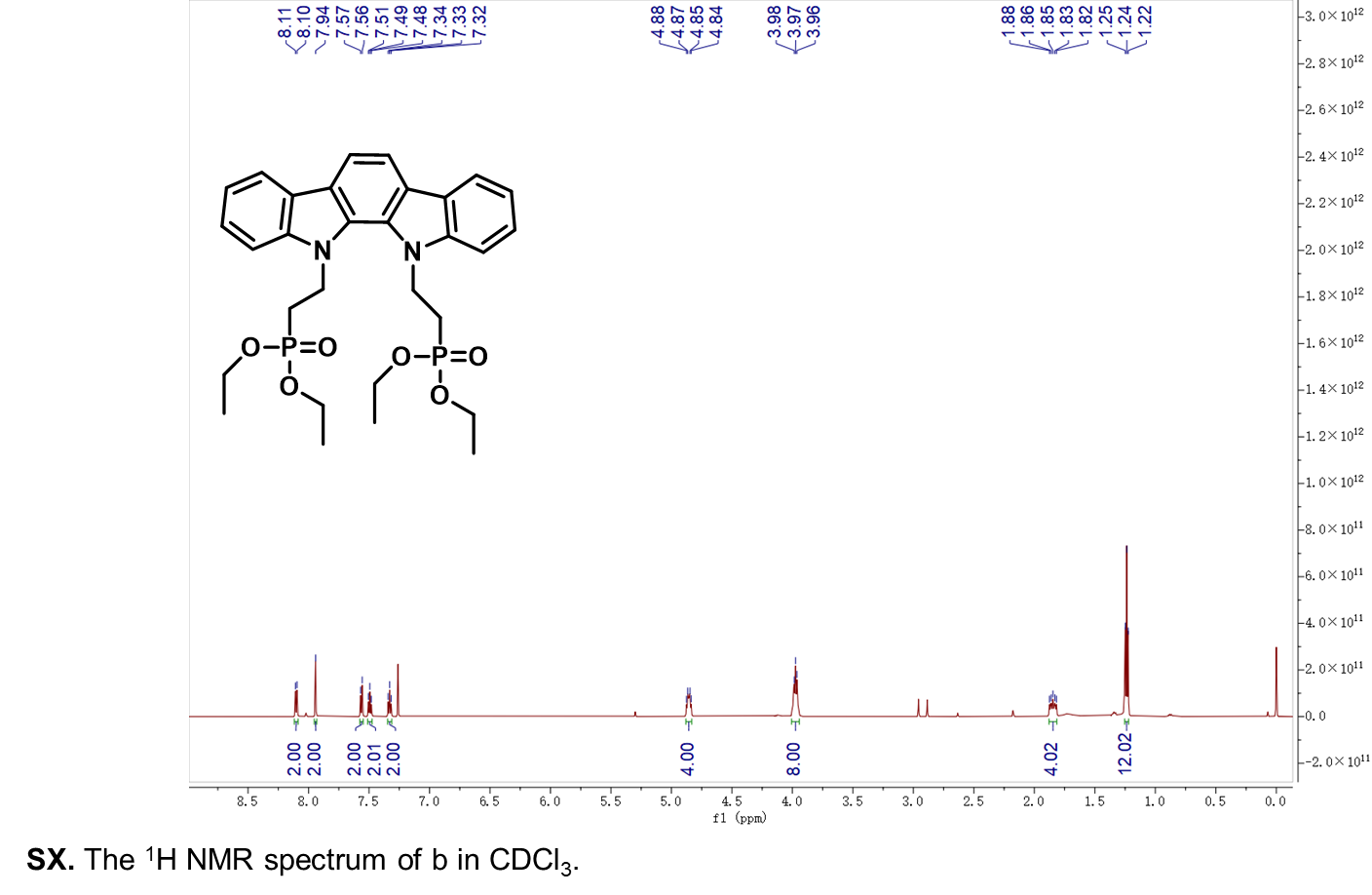


**Figure S33.** The ^1^H NMR spectrum of M2 in CDCl_3_.


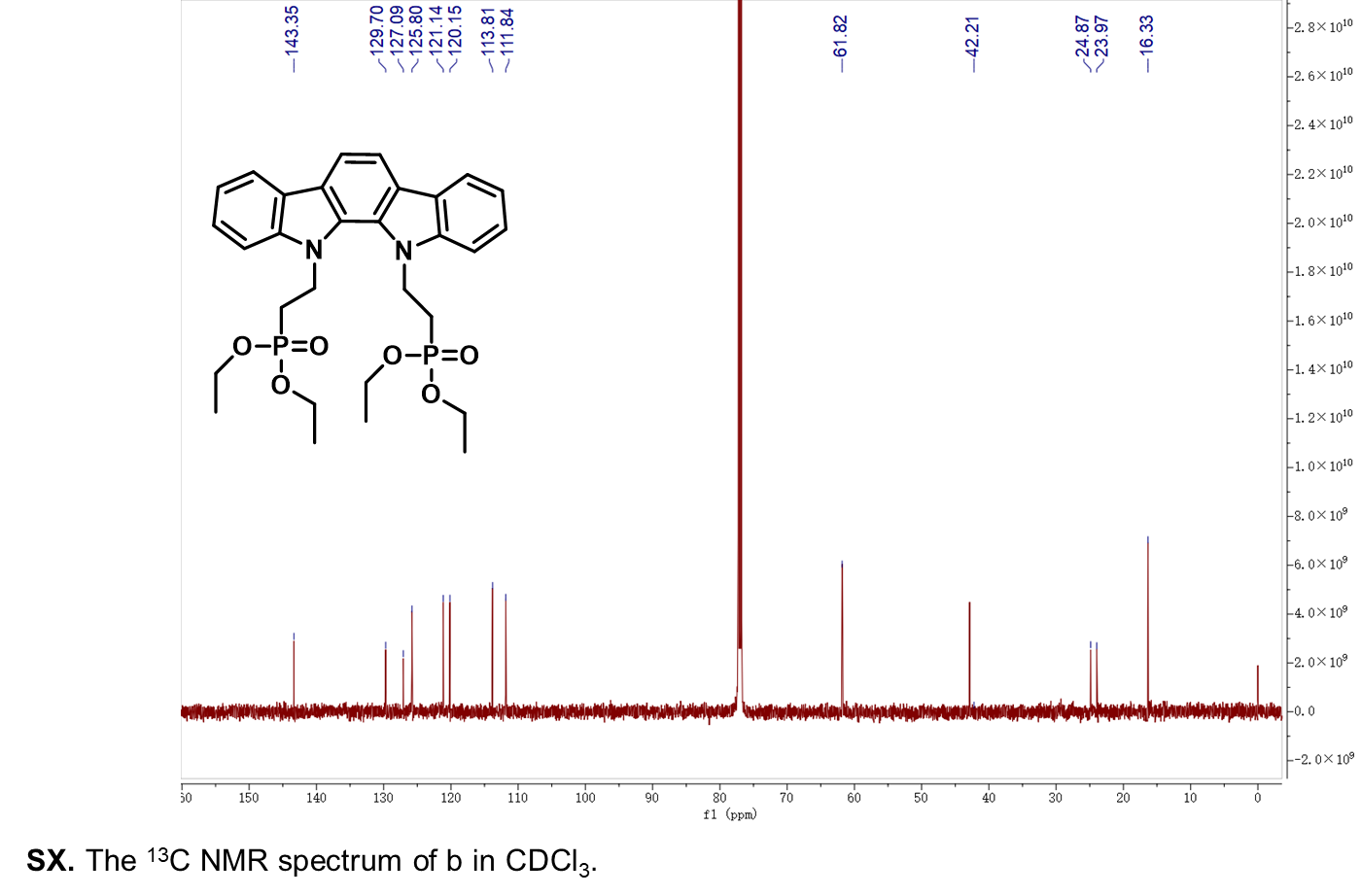


**Figure S34.** The ^13^C NMR spectrum of M2 in CDCl_3_.


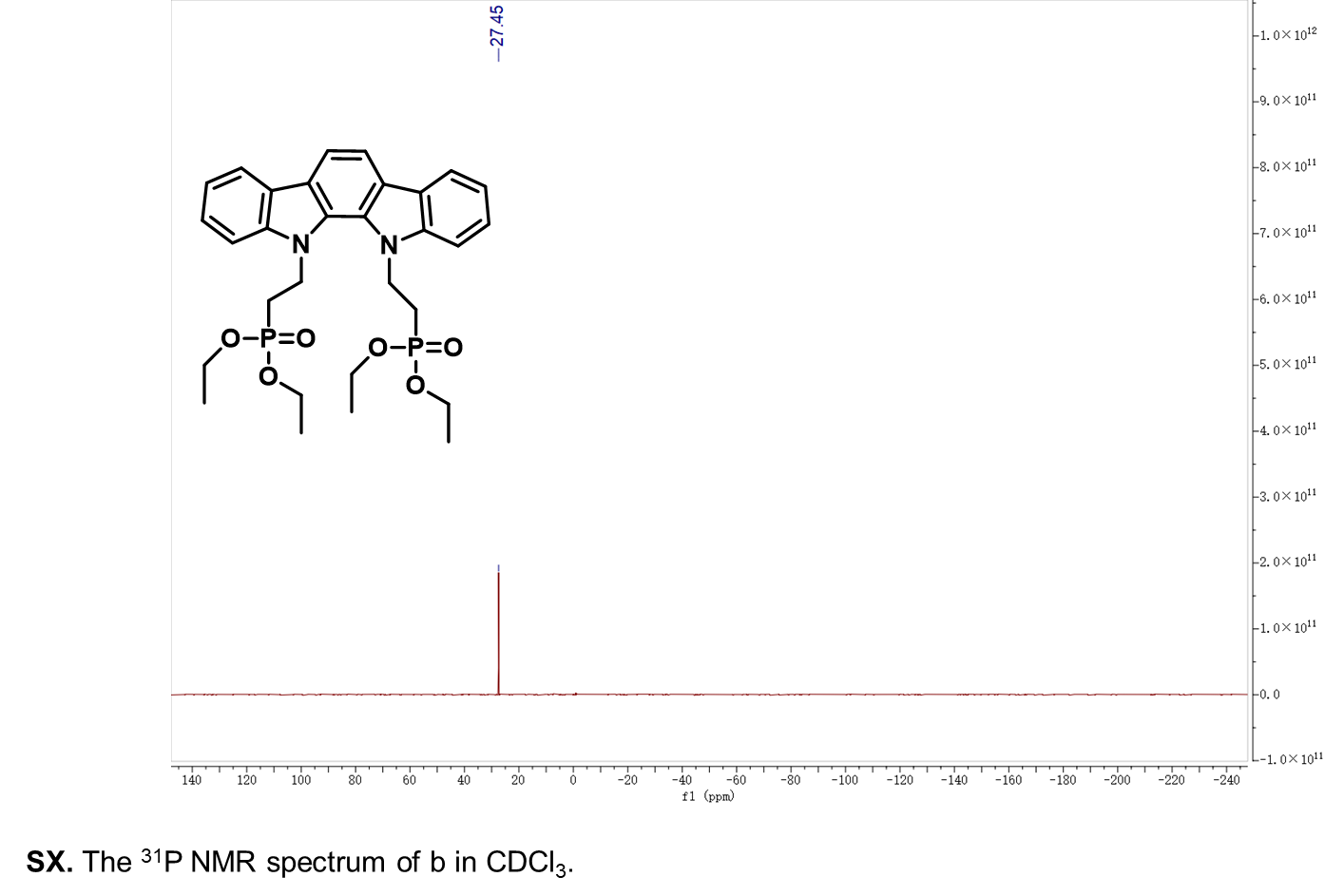


**Figure S35.** The ^31^P NMR spectrum of M2 in CDCl_3_.


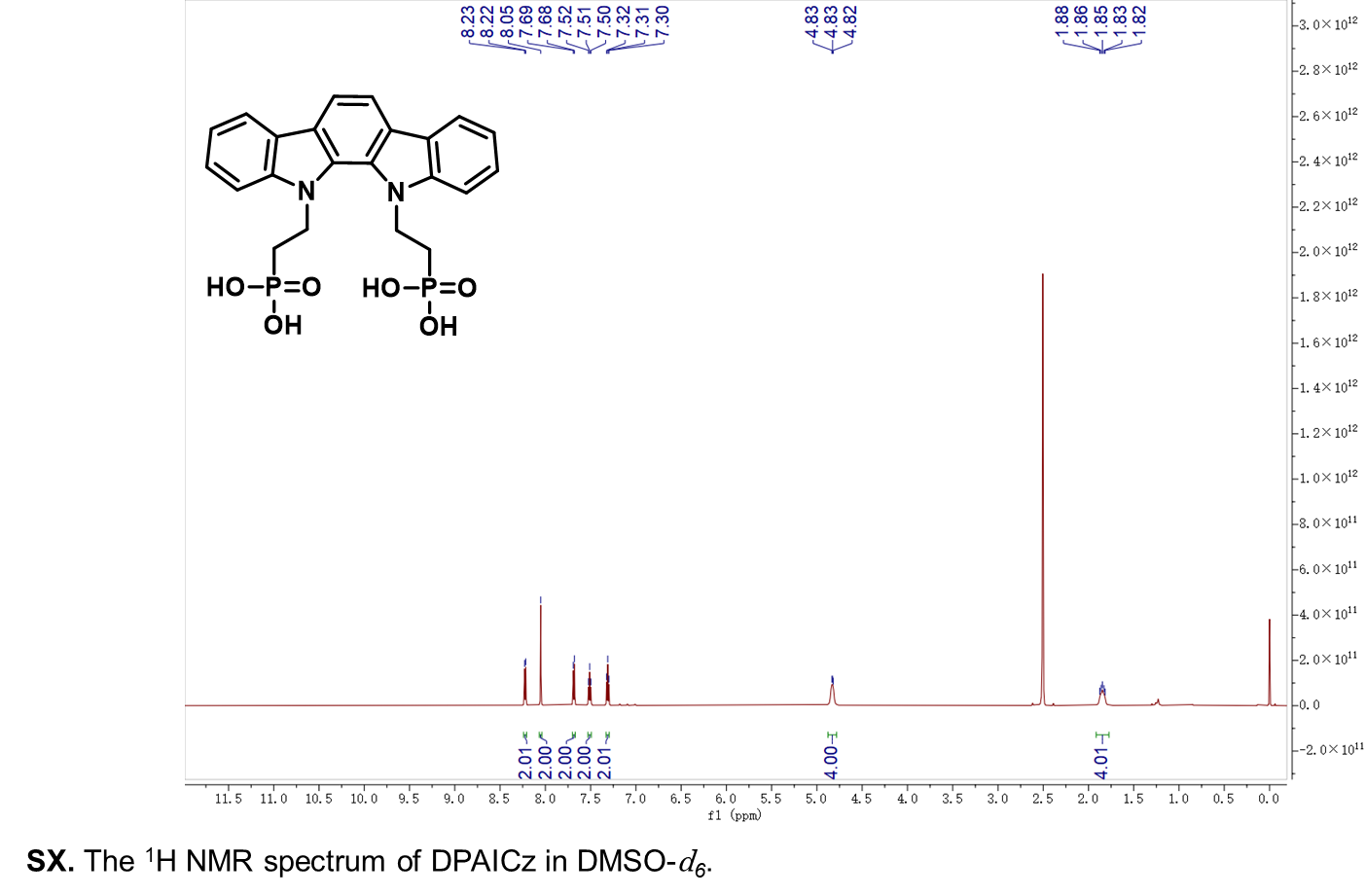


**Figure S36.** The ^1^H NMR spectrum of DPAICz in DMSO-*d_6_*.


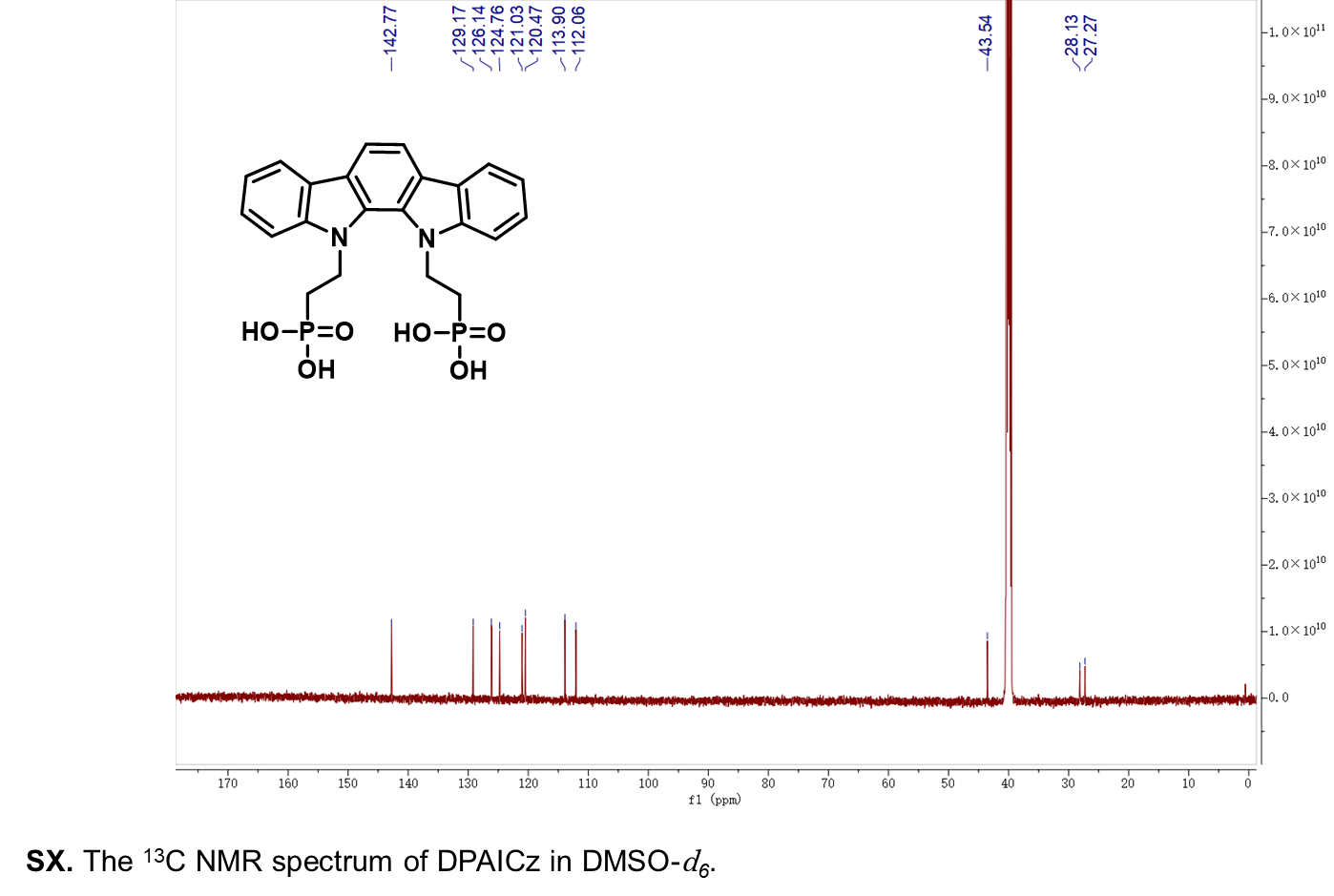


**Figure S37.** The ^13^C NMR spectrum of DPAICz in DMSO-*d_6_*.


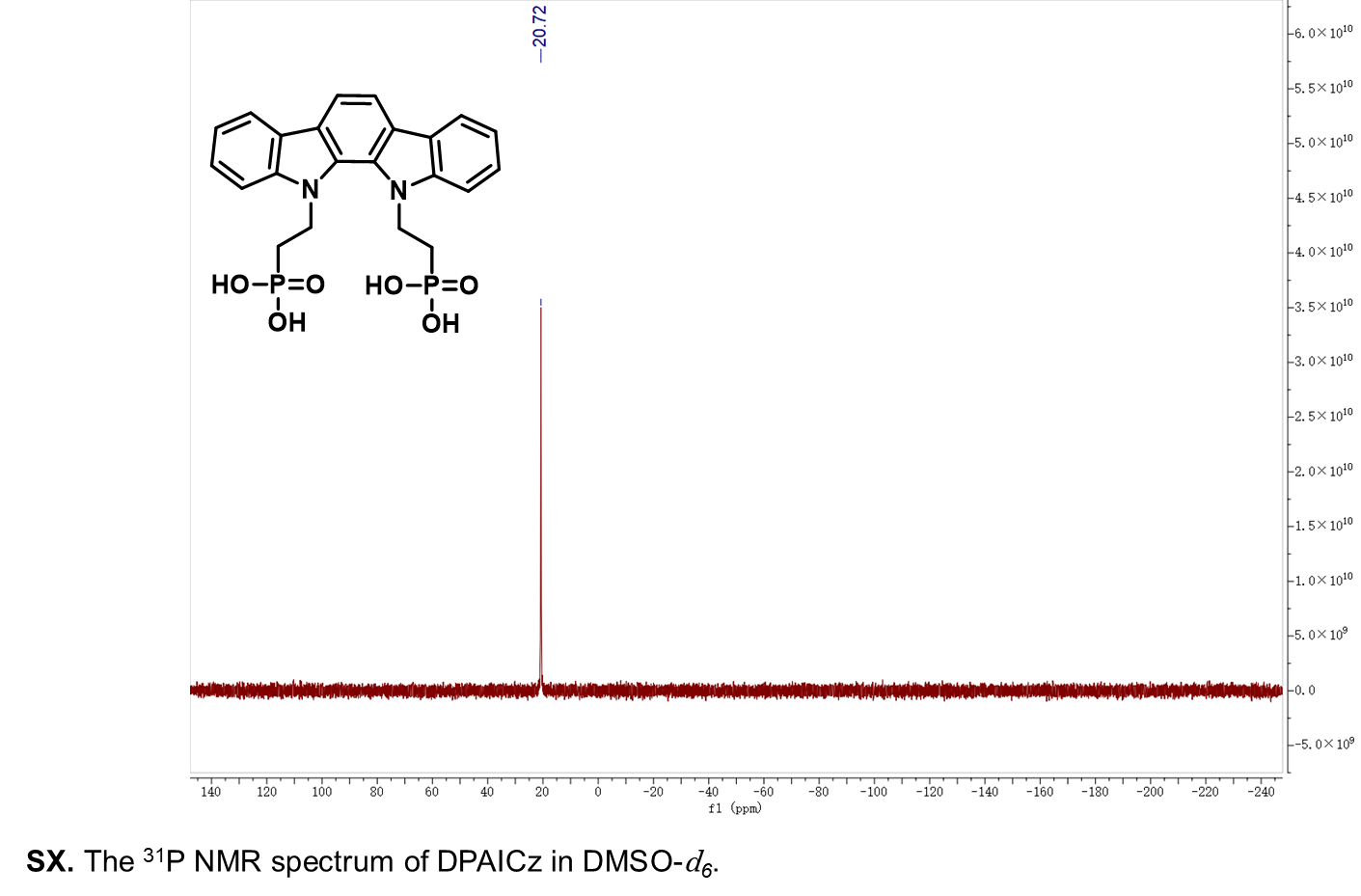


**Figure S38.** The ^31^P NMR spectrum of DPAICz in DMSO-*d_6_*.

**Table S1.** Contact potential distribution (CPD) of 2PACz-coated-ITO and DPAICz-coated-ITO after rinsing with different volume of ethanol.

| Volume of EtOH (μL)  HSL | 0 | 50 | 150 | 250 |
| --- | --- | --- | --- | --- |
| 2PACz | -537.1±21.0 | -446.8±10.4 | -410.7±6.8 | -375.0±10.5 |
| DPAICz | -180.9±2.9 | -202.9±1.7 | -208.5±1.0 | -199.4±2.3 |

**Table S2.** Contact angle of DPAICz deposited on different substrates after rinsing with different volume of ethanol.

| Volume of EtOH (μL)  Substrate | 0 | 50 | 150 | 250 |
| --- | --- | --- | --- | --- |
| Glass/ITO | 72.9±0.2 (°) | 80.4±0.2 (°) | 80.8±0.2 (°) | 79.8±0.2 (°) |
| Textured silicon/ITO | 90.7±0.2 (°) | 96.3±0.5 (°) | 95.9±0.4 (°) | 96.3±0.3 (°) |

**Table S3.** Photovoltaic parameters of single-junction wide-bandgap perovskite solar cells of 1.68 eV derived from *J*-*V* measurements.

| HSL | *V*_OC_ (V) | *J*_SC_ (mA cm^−2^) | FF (%) | PCE (%) |
| --- | --- | --- | --- | --- |
| 2PACz | 1.22 | 21.00 | 84.89 | 21.74 |
| DPAICz | 1.25 | 21.62 | 86.67 | 23.42 |

**Table S4.** Photovoltaic parameters of monolithic silicon/perovskite tandem solar cells derived from *J*-*V* measurements.

| HSL | *V*_OC_ (V) | *J*_SC_ (mA cm^−2^) | FF (%) | PCE (%) |
| --- | --- | --- | --- | --- |
| 2PACz | 1.88 | 20.14 | 80.48 | 30.47 |
| DPAICz | 1.94 | 20.15 | 83.26 | 32.55 |
